# MicroRNA-132 regulates salt-dependent steady-state renin levels in mice

**DOI:** 10.1038/s42003-020-0967-4

**Published:** 2020-05-14

**Authors:** Anton Jan van Zonneveld, Yu Wah Au, Wendy Stam, Sharon van Gelderen, Joris. I. Rotmans, Peter M. T. Deen, Ton J. Rabelink, Roel Bijkerk

**Affiliations:** 1 Department of Internal Medicine (Nephrology) and the Einthoven Laboratory for Vascular and Regenerative Medicine, Leiden University Medical Center, Leiden, The Netherlands; 2 Department of Physiology, Radboud University Medical Center, Nijmegen, The Netherlands

**Keywords:** Kidney, Kidney, miRNAs

## Abstract

The body’s salt and fluid balance is regulated by the renin-angiotensin-aldosterone system. Generation of prostaglandin-E2 (PGE2) in a cyclo-oxygenase-2 (COX-2)-dependent manner in the macula densa, the salt-sensing cells of the kidney, plays a dominant role in renin regulation. Here we show that miR-132 directly targets *Cox-2* and affects subsequent PGE2 and renin levels. MiR-132 is induced and reduced by low- and high salt treatment, respectively, in a p38- and ERK1/2-independent and CREB- and salt inducible kinase-dependent manner. Silencing of miR-132 in mice increases macula densa COX-2 expression and elevates PGE2 and renin levels, which are abrogated by the selective COX-2-inhibitor Celecoxib. Furthermore, a low or high salt diet induces and reduces macula densa miR-132 expression, while low salt diet combined with silencing miR-132 further increases renin levels. Taken together, we demonstrate a posttranscriptional regulatory role for salt-dependent miR-132 in fine-tuning the steady-state levels of renin.

## Abstract (web summary)

Anton Jan van Zonneveld et al. study the role of miR-132 in the regulation of renin in the mouse. They demonstrate that miR-132 directly targets Cox-2 and subsequently affects PGE2 and renin levels. They perform a series of salt diet experiments as well as in vivo manipulations and show that miR-132 controls the steady-state levels of renin in a salt-dependent manner.

## Introduction

In the kidney, the juxtaglomerular cells are known for renin synthesis and release, the rate-limiting step in the activation of the renin–angiotensin–aldosterone system (RAAS), which is central to the regulation of renal salt and water conservation and blood pressure^1^. They act in concert with the cells of the macula densa, which sense changes in renal tubular fluid composition through the sodium chloride concentration, mainly via an apical Na-K-2Cl cotransporter (NKCC2)^2^. A state of hypovolemia/low blood pressure results in low tubular salt concentration triggering the distal part of the nephron to initiate a sequence of events to increase renal blood flow and glomerular filtration rate (tubuloglomerular feedback) and induce renin release^2^. One of these dominant renin-inducing signals involves generation of prostaglandin-E2 (PGE2) by the macula densa in a cyclooxygenase-2 (COX-2)-dependent manner, which communicates to the juxtaglomerular cells to synthesize and release renin into the circulation^3^. Homeostatic mechanisms such as the RAAS need to be tightly controlled and depend on feedback mechanisms to ensure proper regulation. However, the (post-transcriptional) molecular mechanisms underlying such feedback regulation remains unknown. MicroRNAs (miRNAs) often function as feedback regulators and fine tuners of biological processes^4–8^ and may provide such candidates for feedback regulation. MiRNAs are currently the most widely-studied class of post-transcriptional regulators and regulate gene expression by destabilizing RNA and/or inhibiting protein translation through interacting with complementary sequences in the 3′ untranslated regions of their mRNA targets. MiRNAs can simultaneously repress multiple genes to directly influence the output of functionally related biological pathways^9^. It was previously described that miR-663 and miR-181 can bind to the 3′ untranslated region of renin and regulate its mRNA levels in vitro, while it was also demonstrated that these miRNAs were decreased in the renal cortex of hypertensive subjects^10^. Two other miRNAs, miR-330 and miR-125b-5p were shown to be important in balancing the smooth muscle phenotype of juxtaglomerular cells^11^. Despite this emerging literature, an in vivo role for miRNAs in the initiation of the RAAS system remains unexplored. In the present study, we identified salt-dependent miR-132 to function as a feedback modulator of renin levels.

## Results

### MiR-132 regulates renin levels

**Fig. 1:**
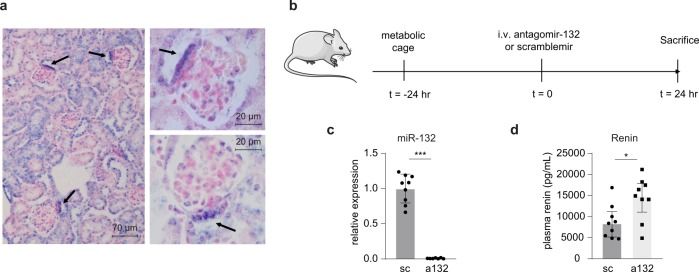
MiR-132 is highly expressed in (distal) tubular structures and controls renin levels. **a** In situ hybridization illustrates that miR-132 is expressed throughout the kidney in mainly tubular epithelial cells, but also interstitial cells and a subset of glomerular cells and particular strong expression is observed in most likely the macula densa (indicated with arrows). Enzymatic development using 4-nitro-blue tetrazolium (NBT) and 5-brom-4-chloro-3′-Indolylphosphate (BCIP) substrate (Roche) forming dark-blue NBT-formazan precipitate was used to visualize miR-132 in combination with nuclear fast red counterstain. **b** Experimental setup, mice were housed on metabolic cages (to collect urine) 24 h before i.v. injection of antagomir-132 or control scramblemir. Twenty-four hours after injection mice were sacrificed. **c** miR-132 expression in the kidney after antagomir-132 treatment (Ct-values decreased from ~27.8 towards ~34.0), as determined by RT-qPCR. Expression is normalized to U6 levels. Data are represented as mean ± SD. **d** miR-132 inhibition results in increased plasma renin levels. Data are represented as median ± interquartile range (IQ1–IQ3), *n* = 7–9. sc scramblemir, a132 antagomir-132, *P < 0.05, ***P < 0.001.

To identify miRNAs with regulatory potential in macula densa cells, a preliminary miRNA profiling was performed of high and low salt treated MMDD-1 macula densa cells which yielded nine miRNAs that were induced or reduced by high salt and vice versa by low salt (Supplementary Fig. 1a), including miR-132. Given our previous interest in miR-132 in renal physiology^12^, we explored whether target genes of miR-132 could have a relation to macula densa function. To that end, all (theoretical and validated) miR-132 targets (derived from www.targetscan.org) were listed and the Ingenuity Pathways Analysis platform was used to list potential upstream regulators of this set of genes. By definition, miR-132 was found as an upstream regulator. But strikingly, an even stronger (statistically) upstream regulator was found in angiotensinogen, the substrate for renin that starts the RAAS system (Supplementary Fig. 1b), implicating that many miR-132 targets (39 genes) are also downstream of angiotensinogen. Furthermore, using in situ hybridization on murine kidney sections, high expression of miR-132 was found in tubular epithelial cells, interstitial cells, and a subset of glomerular cells (Fig. 1a). By far the most prominent expression of miR-132 was observed in the distal tubular epithelial cells neighboring glomeruli, most likely being the cells of the macula densa (Fig. 1a), of which the identity was confirmed by a renal pathologist. Given that the macula densa is the sensor of the kidney to detect changes in tubular salt (reflecting blood salt concentrations) and activates the RAAS system, we hypothesized that miR-132 may be involved in the control of renin synthesis. To investigate this, miR-132 was silenced in wild type mice by i.v. injection of 40 mg/kg antagomir-132 or control scramblemir (*n* = 9 per group). Mice were sacrificed 24 h after the injection (Fig. 1b). Antagomir-mediated silencing of miR-132 in the kidneys was confirmed by RT-qPCR (Fig. 1c; U6 normalized, miR-132 Ct-values decreased from ~27.8 towards ~34.0) and in situ hybridization that demonstrated a marked silencing of miR-132 throughout the kidney (Supplementary Fig. 2). When plasma renin levels were measured in antagomir-132 treated mice, we indeed found a marked increase in renin levels compared to scramblemir control mice (Fig. 1d). In addition, 24 h urine aldosteronuria was determined which demonstrated increased levels in antagomir-132-treated mice (Supplementary Fig. 3).

### Salt-induced miR-132 targets cyclooxygenase-2 (*Cox-2*) and regulates PGE2

**Fig. 2:**
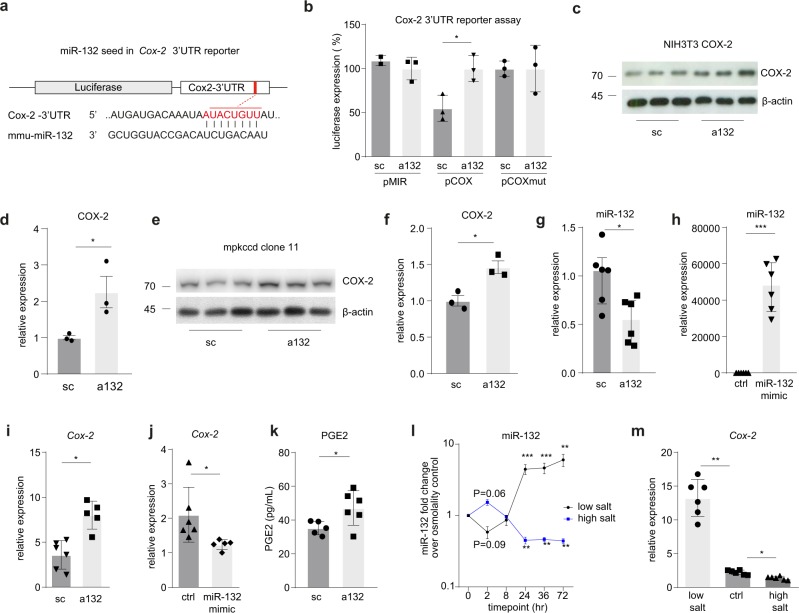
MiR-132 expression is salt concentration dependent and directly targets Cox-2 in macula densa cells. **a** Schematic representation of miR-132 binding site in 3′UTR of Cox-2. **b** Luciferase assay confirms functional, 3′UTR-dependent repression of Cox-2 expression by miR-132. pCOX is Cox2 3′UTR reporter construct, pMIR is empty control reporter construct, pCOXmut is Cox2 3′UTR reporter construct with a single point mutation in the miR-132 binding site, *n* = 2–3 (independent experiments). **c**–**f** Representative western blot for COX-2 in NIH3T3 (**c**) and mpkccd (**e**) cells, with β-actin as housekeeping protein (separately loaded), and corresponding quantification (**d**, **f**) shows increased COX-2 expression following miR-132 silencing. *n* = 3 (independent experiments), numbers adjacent to blots indicate kD. Relative expression is normalized to β-actin and sc set a 1. **g**–**j** Using a macula densa cell line (MMDD-1 cells) we confirmed that miR-132 inhibition (**g**) and miR-132 overexpression (**h**) for 24 h resulted in increased (**i**) and decreased (**j**) Cox-2 gene expression, respectively, as determined by RT-qPCR. MiR-132 is expressed relative to U6, Cox-2 is expressed relative to Gapdh, *n* = 5–6 (independent wells). **k** MiR-132 inhibition increases PGE2 formation after 24 h as compared to scramblemir control treated cells, as determined in the supernatant using ELISA, *n* = 5–6. **l** A low or high salt stimulus of these MMDD-1 cells resulted in initial decreased or increased miR-132 levels followed by increased and decreased, respectively, as determined by RT-qPCR, indicating an active regulatory role for miR-132 upon changes in salt concentration. MiR-132 is expressed relative to U6, *n* = 3 (independent experiments). **m** Cox-2 levels, as determined by RT-qPCR and expressed relative to Gapdh, increase and decrease upon low and high salt treatment, respectively, *n* = 5–6. sc scramblemir, a132 antagomir-132, *P < 0.05, **P < 0.01, ***P < 0.001, data are represented as mean ± SD, except for **g** that is represented as median ± interquartile range (IQ1–IQ3).

Since renin release by the juxtaglomerular cells in the walls of the afferent arteriole of the glomerulus is induced by prostaglandin-E2 (PGE2) generated by cyclooxygenase-2 (COX-2) in the macula densa cells in conditions of hypotension, we next sought to investigate how miR-132 could be involved in this regulation. Using online available algorithms (www.microrna.org) miR-132 was identified as a potential post-transcriptional regulator of *Cox-2* (Fig. 2a). To investigate whether miR-132 could regulate *Cox-2* mRNA expression through the putative binding site, the 3′UTR of *Cox-2* was cloned into a luciferase mRNA stability reporter construct (pCOX). Subsequently we transfected renal epithelial mIMCD3 (IMCD) cells, which endogenously express miR-132, with this construct and control vector (pMIR). As shown in Fig. 2b, treatment of the transfected cells with antagomir-132 increased luciferase expression in pCOX transfected cells, but not in pMIR control cells, indicating that miR-132 attenuates *Cox-2* mRNA expression. To substantiate the miR-132 regulating role of *Cox-2* mRNA and to test its cell-type independency, the effect of antagomir-132 was also tested in NIH3T3 fibroblasts and mouse collecting duct (mpkCCD) cells, which endogenously express *Cox-2* and miR-132. It was found that antagomir-132 treatment increased COX-2 protein levels in both cell types (Fig. 2c–f). Next, using a mouse macula densa cell line (MMDD-1 cells) it was demonstrated that miR-132 mediates *Cox-2* expression as miR-132 inhibition and miR-132 overexpression (Fig. 2g, h) resulted in increased and decreased *Cox-2* gene expression, respectively (Fig. 2i, j). MiR-132 inhibition subsequently led to increased PGE2 secretion by the cells (Fig. 2k). Furthermore, upon a low or high salt stimulus of these MMDD-1 cells, time-dependent changes in miR-132 were observed (Fig. 2l); high salt initially increased miR-132 expression, as compared to mannitol treated control cells, but decreased after 24 h, while the opposite occured with low salt treatment. *Cox-2* expression increased and decreased upon low and high salt, respectively (Fig. 2m). Given this parallel regulation of miR-132 and *Cox-2* by salt treatment, while miR-132 inhibits *Cox-2*, this suggests a feedback role for miR-132.

### Salt-dependent miR-132 is regulated by CREB and salt inducible kinases

**Fig. 3:**
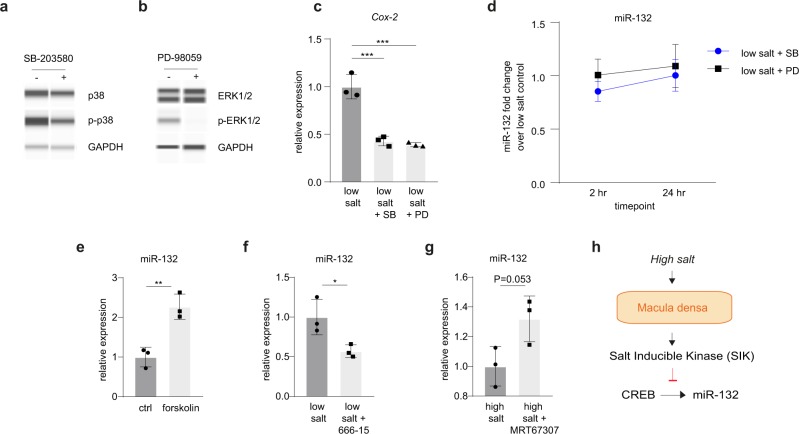
MiR-132 expression is MAPK independent while CREB- and salt inducible kinase-dependent. **a**, **b** p38 inhibitor SB-203580 inhibits p38 activity (**a**), and ERK inhibitor PD-98059 inhibits ERK1/2 activity (**b**), as demonstrated by western blot for total p38 and ERK1/2, as well as phosphorylated p38 (p-p38) and phosphorylated ERK (p-ERK1/2). GAPDH is used as household gene, representative images of *n* = 3. **c** low salt-induced Cox-2 gene expression is inhibited by p38 and ERK inhibition after 24 h, as determined by RT-qPCR. Cox-2 expression is normalized to Gapdh. **d** p38 and ERK inhibition does not affect miR-132 expression after 2 or 24 h under low salt conditions. **e** CREB activator Forskolin induces miR-132 expression after 24 h. **f** CREB-inhibitor 666-15 reduces low salt induced miR-132 expression. **g** Salt inducible kinase inhibitor MRT67307 increases high-salt-reduced miR-132 expression. **d**–**g** As determined by RT-qPCR. Mir-132 is normalized to U6. For panels **c**–**g**: *n* = 3 (averages of independent experiments) (**h**) working hypothesis of salt dependent miR-132 expression. sc scramblemir, a132 antagomir-132, SB p38 inhibitor SB-203580, PD ERK inhibitor PD-98059. *P < 0.05, **P < 0.01, ***P < 0.001, data are represented as mean ± SD.

Since low NaCl-stimulated PGE2 release and COX-2 expression was previously demonstrated to be mediated by MAP kinases (p38 and ERK)^13^, we next sought to investigate whether salt regulated miR-132 expression is also MAP kinase dependent. To that end, MAP kinases were inhibited using the p38 inhibitor SB-203580 and ERK inhibitor PD-98059, as confirmed by western blot (Fig. 3a, b). Inhibition of p38 and ERK in the presence of low salt decreased Cox-2 gene expression after 24 h, which confirms MAPK-dependent regulation of Cox-2 (Fig. 3c) as previously described^13^. However, when we assessed the effect of MAP kinase inhibition on miR-132 expression under low salt conditions, as illustrated in Fig. 3d, neither after 24 h (when miR-132 was strongly induced), or after 2 h (when MAP kinases were shown to be increased)^13^, a change in miR-132 expression was observed (although a slight trend towards decreased levels may be visible 2 h after p38 inhibition). These data suggest miR-132 expression is most likely not MAP kinase dependent.

Next, we aimed to explore alternative mechanisms by which salt may regulate macula densa miR-132 expression. Since it has been shown in other cell types that miR-132 expression is induced by cAMP-response element-binding protein (CREB)^14,15^, MMDD1 cells were treated with Forskolin to induce cAMP dependent CREB activity. Indeed, forskolin was found to induce miR-132 in MMDD-1 cells (Fig. 3e). To assess if low salt-induced miR-132 after 24 h is CREB-dependent, MMDD-1 cells were next incubated under low salt conditions in the presence of CREB-inhibitor 666-15^16^. This was found to decrease miR-132 levels (Fig. 3f) confirming CREB-mediated regulation of miR-132. Finally, we hypothesized that salt inducible kinases (SIK) may provide the link between salt and CREB as it was previously demonstrated that SIKs are salt-induced^17–19^ and inhibit CREB activity via phosphorylating and inactivating its cofactor cAMP-regulated transcriptional coactivator (CRTC)^20–22^. Therefore, MMDD-1 were cultured cells under high salt conditions for 24 h (yielding low miR-132 levels, Fig. 2l) in combination with SIK inhibitor MRT67307^23^. This resulted in increased miR-132 levels (Fig. 3g), indicating SIK suppressed miR-132 expression, most likely via CREB, as summarized in our working hypothesis (Fig. 3h).

### MiR-132 regulates renin levels via COX-2/PGE2 in vivo

**Fig. 4:**
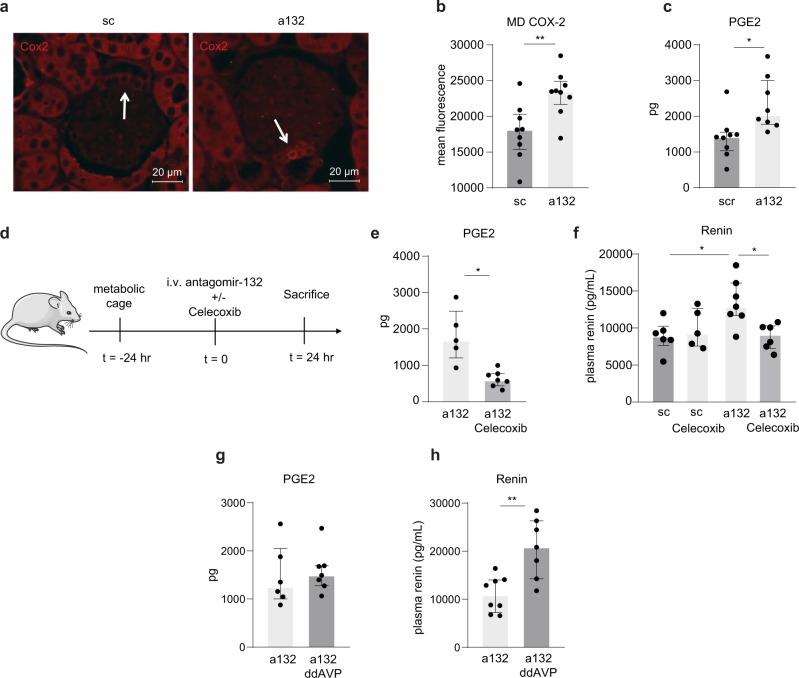
MiR-132 inhibition-mediated renin increase is mediated via COX-2/PGE2 and independent of miR-132 silencing induced diuresis. **a** Representative images of COX-2 staining (**a**) and quantification (**b**) in macula densa cells in scramblemir and antagomir-132 treated mice indicate increased levels due to miR-132 silencing. **c** Renal PGE2 levels (measured in urine using ELISA) subsequently increased due to silencing miR-132. *N* = 8–9 for panels **b**, **c**. **d** Experimental setup, mice were housed on metabolic cages 24 h before i.v. injection of antagomir-132 that was administered in combination with i.p. Celecoxib (or solvent control). Twenty-four hours after injection mice were sacrificed. **e** Renal PGE2 knockdown was confirmed as Celecoxib decreases urinary PGE2 levels, determined by ELISA. **f** COX-2 inhibition with Celecoxib reverses the miR-132 inhibition-induced increase in plasma renin levels, determined by ELISA. *N* = 5–7 for panels **e**, **f**. **g**, **h** antagomiR-132 injection combined with ddAVP administration does not affect urinary PGE2 levels (**g**), while renin levels are increased (**h**), both determined by ELISA, n = 6–8. sc scramblemir, a132 antagomir-132, MD macula densa, *P < 0.05, **P < 0.01, data are represented as median ± interquartile range (IQ1–IQ3).

Next, we sought to investigate the effects of miR-132 silencing on *Cox-2* and PGE2 expression in vivo. Since most renal COX-2 expression is found in the medulla in collecting ducts (Supplementary Fig. 4), we next assessed cortical COX-2 expression and found a trend towards elevated cortical COX-2 levels after miR-132 silencing (Supplementary Fig. 5). Subsequently, macula densa-specific COX-2 staining was quantified (Fig. 4a, b), which demonstrated that systemic inhibition of miR-132, in line with our in vitro observations, resulted in increased levels of COX-2 in the macula densa. Macula densa specificity was confirmed by co-staining COX-2 with NKCC2 (Supplementary Fig. 6). Consequently, PGE2 urine levels were significantly increased in mice, 24 h after antagomir-132 treatment (Fig. 4c). To obtain further support for our hypothesis that COX-2/PGE2-mediated signaling is responsible for the antagomir-132 induced renin levels, mice were treated with antagomir-132 in combination with the selective COX-2 inhibitor Celecoxib (Fig. 4d). PGE2 synthesis was successfully decreased by Celecoxib administration (Fig. 4e). As illustrated in Fig. 4f, COX-2 inhibition by Celecoxib reversed the antagomir-132 induced increase in renin levels, while Celecoxib alone did not alter renin levels, confirming that miR-132 dependent renin levels are mediated by COX-2/PGE2. Of note, Celecoxib treatment did not change urine output, which excludes indirect effects via volume changes. Importantly, we previously found that silencing miR-132 caused weight loss (~0.5 g) and resulted in acute diuresis by inhibiting hypothalamic AVP production subsequently resulting in increased plasma osmolality, decreased urine osmolality and hypovolemia^12^ (see also Supplementary Table 1). To exclude secondary effects on PGE2 and renin levels caused by this, ddAVP was administered which reversed these miR-132 mediated aquaretic effects^12^. Urinary PGE2 remained elevated (Fig. 4g) while plasma renin levels were even further elevated (Fig. 4h), indicating that miR-132 mediated PGE2/renin signaling is independent of miR-132-antagonist induced diuresis.

### Macula densa miR-132 expression is salt dependent in vivo

**Fig. 5:**
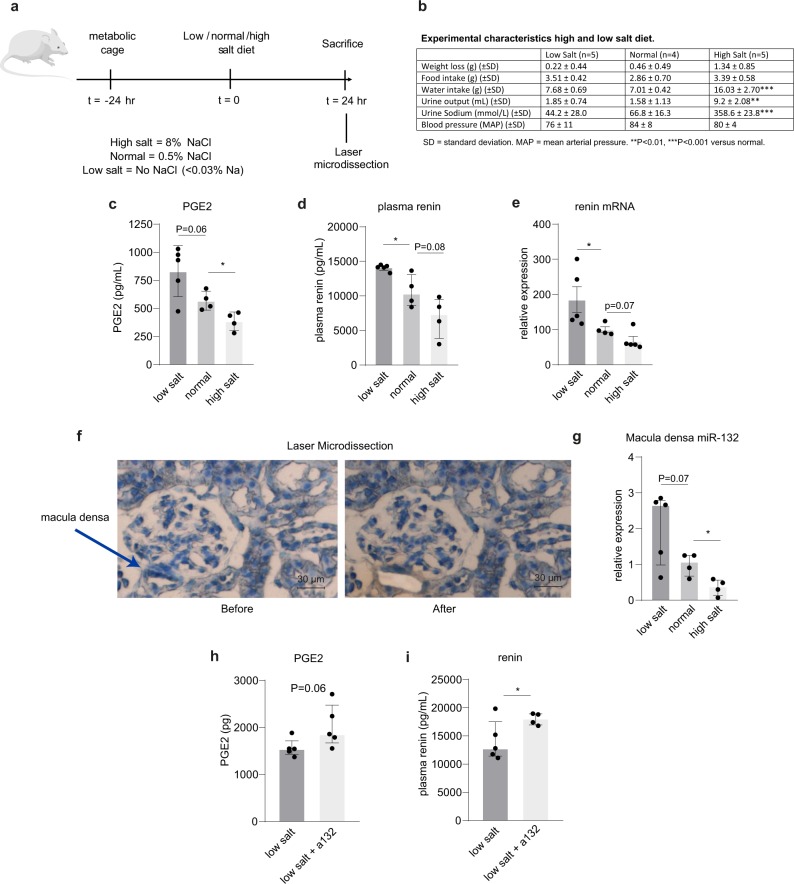
The effect of high and low salt diet on miR-132 expression in the macula densa in vivo. **a** Experimental setup of high and low salt diet. **b** Experimental characteristics of the mice after high, low and normal salt diet. **c** Prostaglandin E2 levels in urine as measured by ELISA. **d** Plasma renin levels as measured by ELISA. **e** Total kidney *renin* mRNA levels, as determined by RT-qPCR, normalized to *Gapdh*. **f**, **g** Representative images (**f**) of a laser microdissected macula densa that was subsequently processed for RNA isolation and miR-132 RT-qPCR, which is depicted in **g**, demonstrating that macula densa miR-132 expression is salt dependent. MiR-132 was not detectable in dissected glomeruli. *N* = 4–6 for panels **c**–**g**. **h**, **i** Mice that were given a low salt diet in combination with silencing of miR-132 displayed a trend towards higher PGE2 (**h**) and increased plasma renin (**i**) levels than low salt diet alone, determined by ELISA, *n* = 4–5. *P < 0.05. Data are represented as median ± interquartile range (IQ1–IQ3).

**Fig. 6:**
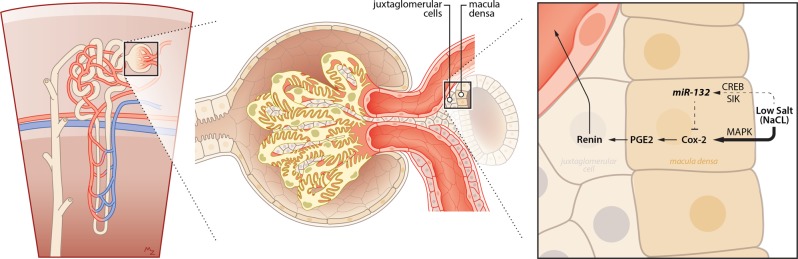
Graphical abstract of microRNA-132 mediated feedback regulation of renin levels. A single nephron (left panel) and a zoomed image of a glomerulus (middle panel) illustrates the closely attached juxtaglomerular cells and the macula densa cells. Right panel: in the macula densa cells of the kidney, low salt (sodium chloride) levels induce Cox-2 via MAP kinases and miR-132 via CREB and salt-inducible kinases. Higher miR-132 levels in the macula densa provides negative feedback and attenuates low-salt induced Cox-2 resulting in tempered synthesis of PGE-2 and subsequently renin.

To investigate the physiological relevance of miR-132 in the regulation of renin levels, we investigated the impact of high and low salt diet on the expression of miR-132 in the macula densa. Mice were given low salt containing chow (no NaCl added, yielding <0.03% Na), normal chow (~0.5% NaCl) or high salt containing chow (8% NaCl) for 24 h (Fig. 5a illustrates the experimental setup). As expected, water intake and urine output were strongly increased in mice on a high salt diet (Fig. 5b). Next, we assessed whether PGE2 and renin levels changed according to their diet. Figure 5c–e confirmed increased PGE2 and plasma renin levels (as well as renin gene expression levels) upon low salt diet, while high salt diet resulted in decreased PGE2 and renin levels, as compared to mice on a normal diet. Finally, miR-132 levels in the macula densa were determined. To that end, laser microdissection was performed on frozen kidney sections to isolate ~10 macula densas per kidney, as illustrated in Fig. 5f. This was followed by RNA isolation and miR-132 RT-qPCR. Low salt diet increased the expression of macula densa miR-132, while high salt diet decreased the expression of the microRNA (Fig. 5g, both compared to normal diet). As a control, also glomeruli were isolated from these sections (with similar surface areas as 10 macula densas), where miR-132 could not be detected. Furthermore, total kidney miR-132 levels did not show a salt sensitive response, although a trend towards increased levels was observed by a high salt diet (Supplementary Fig. 7). These data demonstrate that a low salt diet increases PGE2 and renin levels, as well as miR-132 (in line with in vitro data; Fig. 2l). However, since we identified miR-132 as a negative regulator of the Cox-2/PGE2/renin axis, this suggests that miR-132 provides negative feedback regulation. To further investigate this, mice were given a low salt diet in combination with antagomiR-132. If miR-132 indeed acts as a negative feedback regulator, PGE2 and renin should even further increase. Indeed, Fig. 5h, i demonstrated increased renin levels, and a trend towards increased PGE2 levels, due to miR-132 silencing and low salt diet as compared to low salt diet alone. Taken together, these data support our hypothesis that the expression of miR-132 is salt sensitive and serves as a negative feedback regulator of COX-2/PGE2-mediated control of renin levels (a graphical abstract can be found in Fig. 6).

## Discussion

In this study we identified a role for miR-132 in fine-tuning renin levels via targeting *Cox-2*. We found miR-132 levels to be dependent on salt concentration, suggesting miR-132 plays an active post-transcriptional regulatory role in salt and water handling by the kidney. As such, miR-132 is an important regulator of electrolyte balance and blood volume homeostasis. Our data indicate that miR-132 provides a feedback loop, as low salt induced miR-132 expression, but simultaneously stimulated renin synthesis, while knockdown of miR-132 also resulted in increased, COX-2/PGE2-mediated, renin levels. Moreover, this feedback function of miR-132 was demonstrated as mice that were given low salt diet in combination with antagomir-132 displayed a further increase in plasma renin levels (and a trend towards increased PGE2 levels). This suggests miR-132 serves as an important fine tuner in the tightly controlled regulation of renin levels, such that the system can be balanced within subtle ranges. Importantly, emphasizing its physiological relevance, miR-132 has been demonstrated to be highly increased in kidneys of rats with hypertension^24^.

**Fig. 7:**
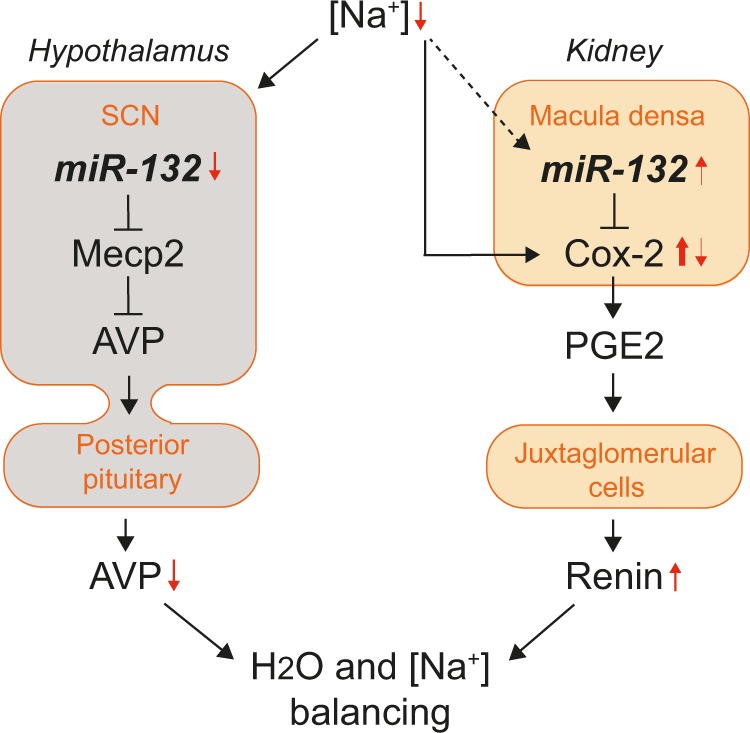
Schematic overview of the role of miR-132 in the body’s osmoregulation. Decreased plasma sodium levels reduce miR-132 expression in the suprachiasmatic nucleus (SCN) of the hypothalamus while decreased tubular sodium induces COX-2 and miR-132 expression in the macula densa cells of the kidney. In the hypothalamus, miR-132 represses *Mecp2* resulting in derepression of AVP transcription. In the case of low miR-132 this yields lower AVP levels that subsequently can no longer be released from the posterior pituitary in the circulation and stimulate water reabsorption in the kidney. At the same time, higher miR-132 in the macula densa provides negative feedback and partially represses low-salt induced *Cox-2* resulting in tempered synthesis of PGE-2. Subsequently, renin synthesis or release from the juxtaglomerular cells is tempered thereby modulating renin–angiotensin–aldosterone system (RAAS) activation that eventually may cause sodium retention and normalization of blood volume.

We previously found miR-132 to regulate water balance through MECP2-mediated vasopressin production in the hypothalamus^12^. Consistent with the findings in the macula densa, miR-132 in the hypothalamus was also regulated by high salt levels. These data suggest miR-132 is a central coordinator and linking pin in the process of salt and water handling by simultaneously regulating vasopressin and renin levels (summarized in Fig. 7). This underlines the concept that single miRNAs can potentially target a set of mRNAs thereby coordinating multiple functionally related pathways that work together to drive developmental, reparative or physiological processes^9,25–27^. In addition, miRNAs are thought to repress these genes to a modest degree and provide feedback mechanisms, making miRNAs fine-tuners of protein output^5,7,28^. This is also true in our study, *Cox-2* is only moderately affected, in contrast to e.g., an approach where aldosterone synthase is knocked out in mice, which showed a ~6-fold increase of COX-2 expression^29^. Nonetheless, it may be very well possible that besides *Cox-2*, miR-132 targets additional genes that are involved in renin synthesis and release (but not related enzymes Cox-1 and mPGES-1, as they do not contain binding sites as determined on targetscan.org and micorna.org). In this context, it is noteworthy that a direct crosstalk between miR-132 and angiotensin II and ACE regulation exists^30,31^, providing a possible additional feedback mechanism for homeostatic control of renin expression since angiotensin II induces miR-132 expression^32^. Furthermore, while we focused on miR-132 in this study, also other miRNAs could very well be involved in *Cox-2* targeting and salt-dependent signaling, as often pathways are regulated by multiple miRNAs^33^, and as also suggested by our pilot miRNA profiling of salt-treated MMDD-1 cells (Supplementary Fig. 1). Although our data describe a macula densa-centered mechanism, miR-132 is strongly expressed in other cell types as well, including proximal tubular epithelial cells, collecting duct (that also express COX-2 and renin) and vascular cells. Given our systemic silencing of miR-132, this suggests possible involvement through these cell types as well. The same applies to the systemic use of Celecoxib for COX-2 inhibition, which may affect multiple cell types. For example, we previously found miR-132 inhibition to affect NHE3 (sodium–hydrogen exchanger 3) expression^12^, which could result in an effect on sodium reabsorption, which subsequently may affect sodium delivery to the macula densa or sodium excretion that alters renin release. Furthermore, a role for miR-132 in arterial cells has been previously described in relation to hypertension^24^, which may translate to altered signaling to the juxtaglomerular apparatus (JGA) impacting renin levels. As such, it may very well be possible that macula densa-independent and JGA-independent effects contribute to the in vivo miR-132 inhibition-induced increase in COX-2/PGE2 and renin. Nonetheless, given that COX-2 was increased in macula densas as a result of miR-132 silencing, while also our salt diet experiments indicate physiological relevance of miR-132 in the macula densa, our data suggest at least a partial direct macula densa and JGA mediated effect.

In our in vitro studies, we demonstrated that miR-132 directly regulates *Cox-2* expression by performing both knockdown and overexpression studies. However, miR-132 mimic treatment resulted in a ~50,000 fold increase in miR-132 levels, while *Cox-2* levels subsequently only decreased 50%. The discrepancy is likely explained by the fact that the RNA-induced silencing complex (RISC; the complex that incorporates the miRNA and together recognize and cleave the target mRNA), becomes saturated and maximal miR-132 loading has been reached, although off-target effects of excess miR-132 in the cell however cannot be excluded. Then, we aimed to explore the mechanism by which salt regulates miR-132 expression. Since low NaCl-stimulated COX-2/PGE2 synthesis was previously demonstrated to be mediated via the MAP kinases p38 and ERK^13^, we investigated whether salt (NaCl) also regulated miR-132 expression via MAP kinases. Given that these effects were previously ascribed to chloride^13^, we assume the effects observed here are also chloride-mediated, although further studies might be necessary to dissect Na and Cl-specific effects. The use of MAP kinase inhibitors however did not affect miR-132, suggesting MAP kinase independent regulation of miR-132 by salt. In contrast, we observed miR-132 expression to be CREB-dependent, and mediated via salt inducible kinases (SIKs). SIKs are induced by salt^17–19^ and inhibit CREB activity via inactivation of CRTCs^20–22^, thereby posing a possible direct mechanism via which miR-132 is regulated by salt. Interestingly, SIK inhibition was previously found to increase miR-132 expression in pancreatic beta cells^34^ and act predominantly in the proximal tubule and the thick ascending limb of the loop of Henle in the kidney^17^. Nonetheless, SIKs were also identified as mediators of the CREB activation cascade downstream of PGE2^23,35^, suggesting miR-132 regulation could also be downstream of PGE2. Furthermore, the SIK inhibitor we used, MRT67307, is not fully specific for SIK as it also inhibits TBK1, MARK1-4, IKKε, and NUAK1, so we cannot exclude that the observed effect is (partly) caused via inhibition of these other kinases, and as such warrants further investigation.

Given our previous finding that silencing miR-132 caused an acute diuresis resulting in increased plasma osmolality and hypovolemia^12^, we sought to determine whether the increase in PGE2 and renin may have been secondary to these physiological triggers. To that end, we administered ddAVP which reversed the miR-132 mediated diuretic effects and normalized plasma osmolality and hypovolemia^12^, while still yielding miR-132 knockdown. PGE2 levels remained elevated while plasma renin levels did not decrease and were even further augmented, indicating that miR-132 directly affects renin levels via COX-2/PGE2 and this is not secondary to the diuretic response. The reason for the further increase of renin by ddAVP however is unclear although this might be explained by increased synthesis in the collecting duct^36^ as this was previously shown to be induced by ddAVP via the vasopressin type2-receptor (V2R), independently of the RAAS^36^. Nonetheless, since it is unclear, in contrast to mice, whether V2R is also expressed in the distal part of thick ascending limb of henle in humans, these results may not be extrapolated to humans and should be carefully considered.

Another potentially important implication of our findings is that an increase in miR-132 could lead to increased sodium excretion and increased water reabsorption (see also Fig. 7) which could eventually result in hyponatremia. Interesting in this respect are the findings that the occurrence of thiazide-induced hyponatremia is related to tubular PGE2 uptake and release^37^, where an association was identified of thiazide induced hyponatremia with a variant in SLCO2A1, which encodes a prostaglandin transporter in the distal nephron. This led to an increased urinary PGE2 excretion and increased free water reabsorption, at least partly as a consequence of altered tubular PGE2 uptake and release. Given our identified role for miR-132 in COX-2 mediated PGE2 synthesis as well as water reabsorption^12^, it is important to consider a potential role for miR-132 in this process as described for thiazide induced hyponatremia. Furthermore, we did not observe a decrease in renin levels with celecoxib treatment in the scramblemir control group, which is a usual finding with NSAIDs^38^. Although speculative, this might be related to the timing of our experiment, as most literature appears to indicate lower renin levels due to NSAIDs after longer durations of administration than the 24 h of our study^38^.

While to our knowledge no microRNAs have been described to play a role in macula densa function, the importance of miRNAs in the functionality of juxtaglomerular cells was previously acknowledged, as mice lacking Dicer, the enzyme that produces mature miRNAs, in cells of the renin lineage, have reduced renin levels, decreased blood pressure and impaired kidney development characterized by striking vascular abnormalities and prominent striped fibrosis^39^. Interestingly, mir-132 was shown to be involved in the progression of renal fibrosis by affecting myofibroblast proliferation^40^. In addition, miR-132 expression in the juxtaglomerular cell itself was found to associate with renin expression^11^, hinting at an additional role for miR-132 in the function of the juxtaglomerular cells themselves. Although intriguing, these data indicating a role for miR-132 in several processes, also reveal potential miR-132-based therapeutic targeting problems through both aspecific and off-target effects. Further studies are necessary to determine the therapeutic potential of miR-132-based strategies and to assess the primary effects.

Taken together, our data reveal that miR-132 plays an important fine-tuning role in the physiological regulation of renin levels.

## Methods

### Animals

Male balb/c mice were used for i.v. antagomir-132 administration experiments (8 weeks old, *n* = 8–9 per group, Charles River Nederland, Maastricht, the Netherlands). Twenty-four hours before i.v. tail injection of antagomir-132 or control scramblemir (40 mg/kg bodyweight), mice were individually housed in metabolic cages until day of sacrifice allowing the collection of 24 h urine. For the Celecoxib experiment, mice were administered 30 mg/kg i.p. of celecoxib in a mixture of 10% DMSO/ 90% sunflower oil or vehicle control. For the ddAVP experiment, archival materials were used from our previous study^12^. Standard chow diet and drinking water were provided ad libitum. For the salt diet experiment, 8 weeks old mice were fed low salt containing chow (no NaCl added, yielding <0.03% Na), normal chow (~0.5% NaCl) or high salt containing chow (8% NaCl) (Snniff Spezialdiäten, Soest, the Netherlands) for 24 h. Blood pressure was assessed using the CODA noninvasive tail cuff system (Kent Scientific, Torrington, CT) in conscious mice. The final blood pressure value was averaged from ten consecutive measurements for each mouse. Blood was drawn by cardiac puncture and blood plasma was obtained by centrifugation (15 min at 3000 rpm at room temperature). The animal welfare committee of the Leiden University Medical Center approved all animal experiments and protocols.

### Cell culture

The cell lines NIH3T3 and mIMCD3 (IMCD) were obtained from ATCC (American Type Culture Collection, Manassas, VA). NIH3T3 cells were maintained in Dulbecco’s modified Eagle medium (DMEM, Gibco/Invitrogen, Breda, the Netherlands), and IMCD cells in DMEM/HAM-F12 medium. Both media were supplemented with 10% fetal calf serum (Bio Whittaker/Cambrex, Verviers, Belgium) and 1× l-glutamin (Invitrogen) at a final concentration of 2 mM. Mouse cortical collecting duct (mpkCCD) cells (clone 11) were grown as previously described^41^ in a modified defined medium (DMEM:Ham’s F12 1:1 vol/vol; 60 nM sodium selenate, 5 μg/ml transferrin, 2 mM glutamine, 50 nM dexamethasone, 1 nM triiodothyronine, 10 ng/ml epidermal growth factor, 5 μg/ml insulin, 20 mM d-glucose, 2% fetal calf serum and 20 mM HEPES (pH 7.4). MMDD-1 cells were kindly provided by Dr. Bachmann (Charité-Universitätsmedizin Berlin, Germany) and maintained in DMEM/HAM-F12 medium supplemented with 10% fetal calf serum. High salt treatment of MMDD-1 cells was performed by adding NaCl (7.305 mg/ml) to culture media with a final osmolality of 550 mOsm/kg, while control cells were treated with mannitol (final osmolality 550 mOsm/kg, to control for increased osmolality). Low salt treatment was performed by combining culture media (300 mOsm/kg) 1:1 with 300 mM mannitol, reducing salt content to 50%, while not changing osmolality. To control for the dilution of medium, we included a control where we combined culture media 1:1 with (300 mOsm/kg) saline. Forskolin was used at a final concentration of 10 μM (R&D systems, Minneapolis, MN, USA), CREB-inhibitor 666-15 at 0.5 μM and (R&D), salt-inducible kinase inhibitor MRT67307 at 2 μM (R&D), ERK1/2 inhibitor PD-98059 (10 μM) (New England Biolabs, Ipswich, MA, USA) and p38 inhibitor SB-203580 at 10 μM (Abcam).

### MiRNA profiling pilot and pathway analysis

For miRNA cDNA synthesis, total RNA was reverse transcribed using the miRNA reverse transcription kit (Applied Biosystems, Foster City, CA) in combination with the stem-loop Megaplex Human primer pools A V2.1 (Applied Biosystems) according to manufacturer’s instructions. For each cDNA sample, 384 micro-RNAs including six controls (RNU44, RNU48, 4*U6), were profiled using TaqMan Array MicroRNA Human Card A V2.0 (Applied Biosystems) according to manufacturer’s instructions. All arrays were run on a 7900HT Fast Real-Time PCR System (Applied Biosystems) and used default thermal-cycling conditions. For each array, the obtained cycle threshold values were converted to relative quantities normalized to U6.

Pathway analyses were performed using Ingenuity Pathway Analysis software (Ingenuity, Qiagen, Redwood City, CA).

### Antagomir design and mimics

Synthesis of cholesterol-conjugated single-stranded “antagomirs”, (Thermo Scientific, Waltham, MA, USA) were described previously^42^. For antagomir-132 the following sequence was used: 5′-c_s_g_s_accauggcuguagacug_s_u_s_u_s_a_s_-Chol-3′. A control “scramblemir” was used, which is generated from a randomized nucleotide sequence which should not bind to any known microRNAs: 5′-a_s_u_s_gacuaucgcuauucgc_s_a_s_u_s_g_s_-Chol-3′. The lower case letters represent 2′-*O*Me-modified nucleotides; subscript “s” represents phosphorothiate linkage; “Chol” indicates a cholesterol-group linked through a hydroxyprolinol linkage. MiR-132 mimics (miRCURY LNA miRNA Mimics, Exiqon, Denmark), double stranded with locked nucleic acids (LNA) incorporated, were transfected with final concentration of 50 nM using Lipofectamine 2000 (Invitrogen).

### Plasma and urine assays

Urinary PGE2 levels were determined using an enzymatic PGE immunoassay (Cayman Chemical), according to the protocol of the manufacturer. Measurements were performed on 24 h urine and normalized to urine volumes, except in Fig. 5c where PGE2 concentration is plotted. Plasma renin levels (Sigma Aldrich, Zwijndrecht, the Netherlands) and urinary aldosterone levels (Abcam, Cambridge, UK) were determined using ELISA according to the protocol of the manufacturer.

### *Cox-2* 3′UTR miR-132 reporter assay

A synthetic, double-stranded oligonucleotide spanning a 60 bp region of the murine 3′ UTR of *Cox-2* mRNA containing the putative miR-132 binding site with or without a single point mutation in the binding site (see Supplementary Methods) was cloned downstream of the firefly luciferase reporter gene in the pMIR-report^TM^ Expression Reporter Vector System (Applied Biosystems, Amsterdam, the Netherlands), thereby generating pMIR-132-report and pMIR-132mut-report. The same construct lacking the miRNA-132 seed sequence (pMIR132-reportNC) served as a negative control. Sequence analysis confirmed proper cloning and sequence of the inserts. A renilla luciferase expression plasmid (pRL-SV40, Promega, Leiden, the Netherlands) served as a transfection efficiency control. Antagomir-132 or control scramblemir (5 mg/ml) was added to a near confluent layer of IMCD cells. Twenty-four hours after antagomir treatment, IMCD cells were trypsinized, resuspended in 500 ml serum-free Optimem culture medium (Gibco), and 1.5 µg pMIR-132-report or pMIR-132-reportNC, together with 150 ng pRL-SV40 were added. The cell suspension was chilled for 10 min at 4 °C and electroporated using the Gene Pulser II (Bio-Rad Laboratories, Veenendaal, the Netherlands). After 10 min recovery time at room temperature, 1.5 × 10^5^ cells were plated in a 24-wells plate in triplicate. After 24 h, the firefly-luciferase and renilla-luciferase signals were measured using a Dual-Luciferase Assay Reporter System (Promega) in a Lumat LB9507 luminometer (EG&G Berthold, Bundoora, Australia).

### Laser microdissection

Five micrometer fixed frozen sections were placed on membrane glass slides (MembraneSlide 1.0 PEN, Carl Zeiss Sliedrecht, the Netherlands) and stained and dehydrated using a Arcturus Histogene LCM frozen section staining kit (Thermofisher Scientific, Waltham, MA, USA) according to the manufacturer’s instructions. The sections were then viewed with the LCM microscope (20× objective; Zeiss PALM Microbeam). The macula densas were identified by their location and morphology and dissected with the laser and captured in the collection tubes. ~10 macula densas (total surface area around ~5000 µm^2^) per kidney were isolated. Subsequently, RNA was isolated as described below.

### Western blot

Western blot was performed on cellular lysates that were harvested in lysis buffer (50 mM Tris-HCl pH 7.5, 150 mM NaCl, 1% SDS, 0.5% deoxycholate, 0,5% Triton X-100) containing protease inhibitors (Complete protease inhibitor cocktail, Roche, Basel, Switzerland). Equal protein amounts were separated using SDS-polyacrylamide gels (SDS-PAGE) under reducing conditions and transferred to a nitrocellulose membrane (Bio-Rad Laboratories, Veenendaal, The Netherlands). Primary antibodies were used to detect β-actin (MABT825; Millipore, Darmstadt, Germany) and COX-2 (ab15191; Abcam, Cambridge, UK). Bound primary antibody was labeled with the appropriate HRP-conjugated secondary antibody in blocking solution. For detection we used chemiluminescent reagents (West Dura supersignal; ThermoFisher Scientific, Waltham, MA, USA) and exposed on Hyperfilm ECL (Amersham). Quantitative analysis of the protein bands was performed using ImageJ software and normalized to β-actin. p38 (Cell Signaling Technology; 9212), phospho-p38 (Cell Signaling Technology; 9211s), ERK1/2 (Cell Signaling Technology; 9102s), phospho-ERK1/2 (Cell Signaling Technology; 9101S) and GAPDH (Cell Signaling Technology; 5174S) were analyzed through capillary western blot (Wes, Protein simple, San Jose, CA) and analyzed using the Compass for SW software (Protein Simple). Full uncropped blots have been included as Supplementary Fig. 8.

### Immunohistochemistry

Paraffin-embedded kidneys were sectioned at 4 µm. After de-paraffinization, antigen retrieval was performed by the heat-induced antigen retrieval method using citrate buffer (10 mM, pH = 6.0) in a microwave for 20 min. Sections were incubated with a specific rabbit antibody against murine COX-2 (ab15191; Abcam) or NKCC2 (LS-C313275-100; LSBio, Seattle, WA, USA) followed by an appropriate secondary antibody labeled with Alexa-568 (A-11011; Molecular Probes, Invitrogen) or horseradish peroxidase (HRP)-conjugated secondary antibodies (Jackson Immunoresearch, Westgrove, PA, USA). Images were made using a Leica DMI6000 microscope (Leica Microsystems, Rijswijk, The Netherlands). Quantification of immunohistochemistry was performed using image J software (NIH, Bethesda, MD, USA); for Fig. 4b, we analyzed ~7 macula densas per kidney section and measured the average fluorescence intensity.

### RNA isolation and RT-qPCR analysis

Total RNA was isolated using Trizol reagent (Invitrogen). MiR-132 expression analysis was performed using Taqman® miR assays (Applied Biosystems, Foster City, CA, USA) according to manufacturer’s instructions. RNU6B was used for normalization (sense U6: CTCGCTTCGGCAGCACA and antisense U6: AACGCTTCACGAATTTGCGT). Two hundred and fifty nanogram of total RNA was reverse transcribed using oligo(dT) primers and M-MLV First-Strand Synthesis system (Invitrogen) according to the manufacturers protocol. Quantitative PCR of target genes was done using SYBR Green Master Mix (Applied Biosystems). Used primer sequences of target genes were: *Cox-2* (sense): TGAGCAACTATTCCAAAC; *Cox-2* (antisense): GCACGTAGTCTTCGATCA; *Renin* (sense): CTCTCTGGGCACTCTTGTTGC; *Renin* (antisense): GGGAGGTAAGATTGGTCAAGGA; *Gapdh* (sense): ACTCCCACTCTTCCACCTTC; *Gapdh* (antisense): CACCACCCTGTTGCTGTAG. Levels of expression were normalized to *Gapdh* and quantified using the delta delta Ct method.

### In situ hybridization

In situ hybridization for miR-132 was performed by Bioneer (Denmark) as previously described^43^. Enzymatic development using 4-nitro-blue tetrazolium (NBT) and 5-brom-4-chloro-3′-Indolylphosphate (BCIP) substrate (Roche) forming dark-blue NBT-formazan precipitate was used to visualize miR-132 in combination with nuclear fast red counterstain (Vector Laboratories, Burlingame, CA).

### Statistics and reproducibility

Results are expressed as mean ± standard deviation (SD) for normally distributed data or median ± interquartile range (IQR; IQ1–IQ3) for data that was not normally distributed. Statistical analysis when comparing two groups was performed using student’s *t*-test, or Mann–Whithey *t*-test when data was not normally distributed (using GraphPad Prism software). For Fig. 4f, to compare multiple groups, we used general linear modeling in SPSS with Sidak post-hoc test. Indicated group sizes (*n*) represents biologically independent samples/experiments. *P*-values <0.05 were considered statistically significant.

### Reporting summary

Further information on research design is available in the Nature Research Reporting Summary linked to this article.

## Supplementary information

Supplementary Information

## Data Availability

The source data underlying the western blots are provided in Supplementary Fig. 8 and the source data underlying the graphs are provided as a separate source data file. Other relevant data are available from the corresponding author upon request.

## Source data

Source Data
Peer Review File
Reporting Summary
