# Supplementary material for: MicroRNA-132 regulates salt-dependent steady-state renin levels in mice
Source: Commun Biol. 2020 May 14;3:238. doi: 10.1038/s42003-020-0967-4 (PMC7224281; doi:10.1038/s42003-020-0967-4)
Supplement: Supplementary file 1 — Supplementary Information [file 42003_2020_967_MOESM1_ESM.pdf]

A

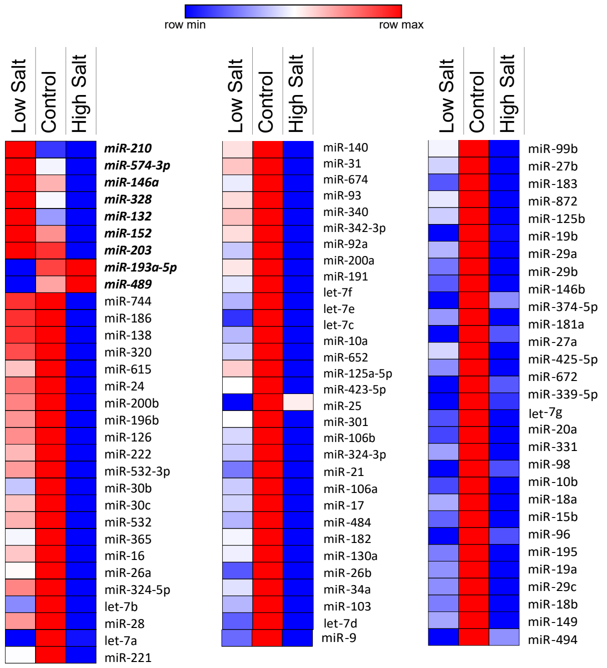

B

Top Upstream Regulators	
Upstream Regulator	p-value of overlap
AGT	4,22E-17
mir-132	1,22E-14
miR-132-3p (and other miRNAs w/seed AACAGUC)	4,01E-09
miR-21-5p (and other miRNAs w/seed AGCUUAU)	6,63E-07
HMG20B	4,94E-06

Supplementary Figure 1. Profiling data indicating a role for miR-132 in macula densa function. (A) Heatmap of miRNA profiling of low, normal and high salt treated MMDD1 cells. Red is high expression, blue is low expression. Cells were incubated for 16 hours. MiRNAs in ***bold italic*** were either increased or decreased by low salt and vice versa by high salt, as compared to controls. (B) Ingenuity Pathway Analysis of all theoretical and validated miR-132 target genes (extracted from targetscan.org) suggests AGT (angiotensinogen) regulates several genes (39) also targeted by miR-132.

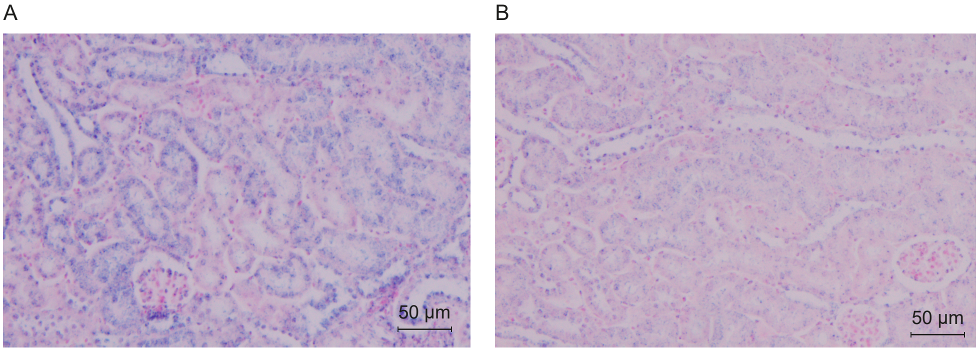

Supplementary Figure 2. *In situ* hybridization demonstrates knockdown of miR-132. Representative images of *in situ* hybridization for miR-132 illustrates strong expression in kidneys from control (scramblemir treated) mice (A) which is decreased in kidneys from antagomir-132 treated mice (B).

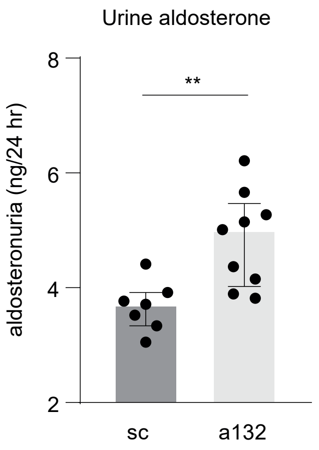

Supplementary Figure 3. Urinary 24 hr aldosterone levels increase after miR-132 silencing. Aldosterone levels in urine as determined by ELISA, n=7-9. sc = scramblemir, a132 = antagomir-132. **P<0.01, data are represented as median ± interquartile range (IQ1-IQ3).

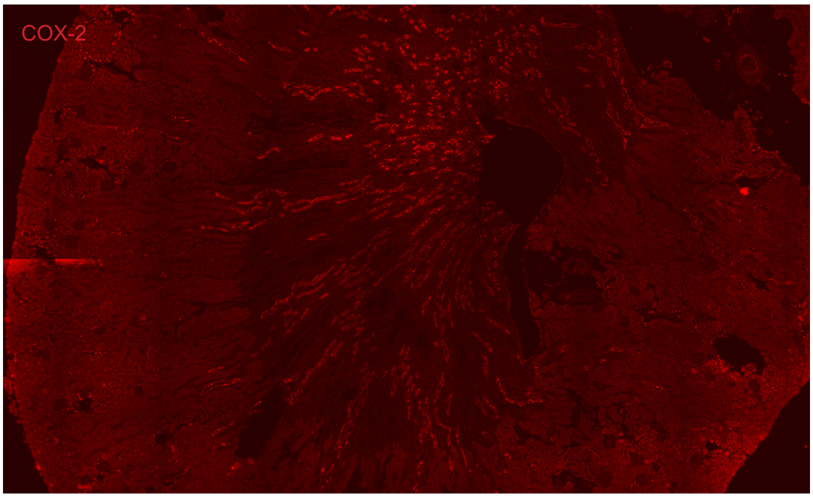

Supplementary Figure 4. Whole kidney image indicates COX-2 is mainly expressed in medulla. Representative image showing COX-2 staining (intense red signal) in a kidney of a scramblemir treated control mouse. Note high red background levels to allow easy visualization of the kidney.

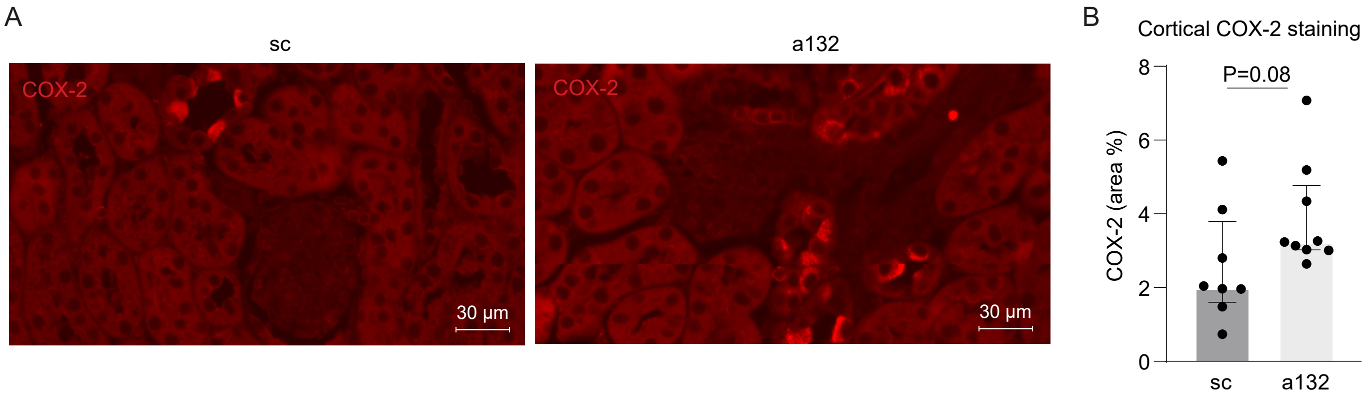

Supplementary Figure 5. MiR-132 inhibition results in increased cortical COX-2 expression. (A-B) Representative images of COX-2 staining (A) and quantification (B) shows a trend towards increased levels in antagomir-132 treated mice, n=8-9. sc = scramblemir, a132 = antagomir-132, data are represented as median ± interquartile range (IQ1-IQ3).

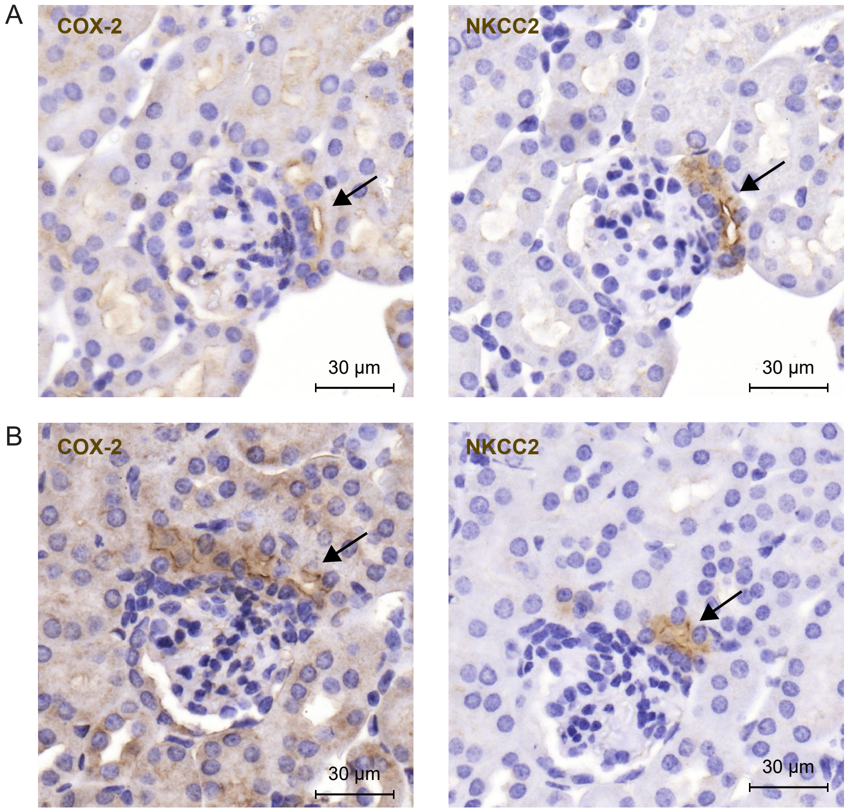

Supplementary Figure 6. Co-staining of COX-2 and NKCC2. (A-B) Representative images of COX-2 and NKCC2 staining in sequential sections (A and B illustrate two examples) indicating double positive structures representing macula densa.

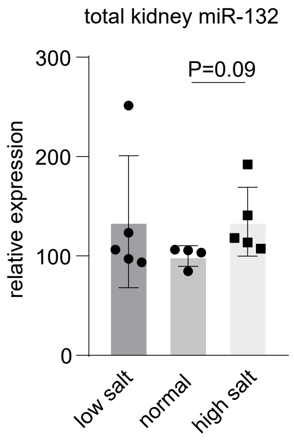

Supplementary Figure 7. Total kidney miR-132 levels after high or low salt diet. U6 normalized, determined by RT-qPCR, n=4-5.

Supplementary Figure 8. Full blots (1)

Full blot COX-2 3t3 cells Figure 2C

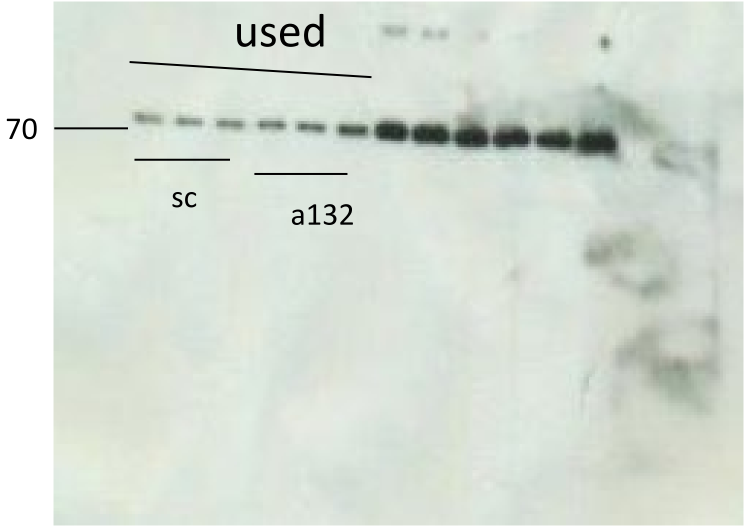

Full blot B-actin 3t3 cells Figure 2C

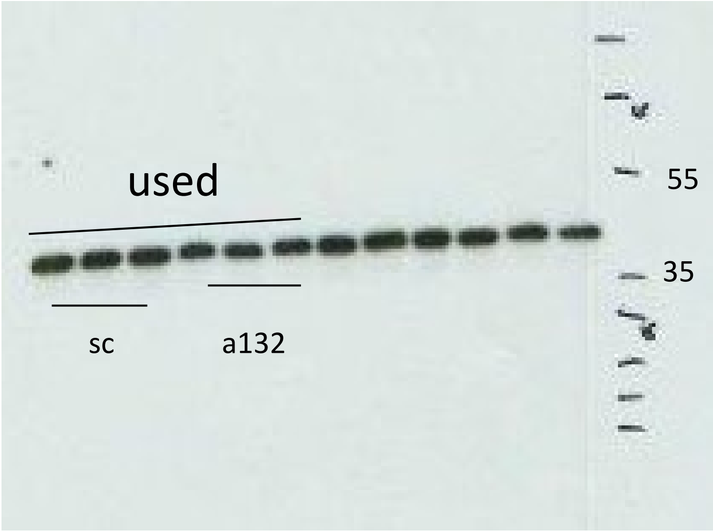

Full blot COX-2 mpkccd cells Figure 2E

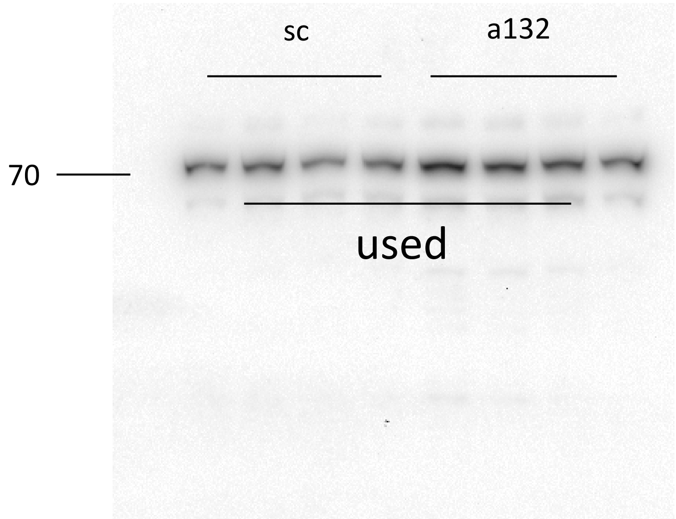

Full blot b-actin mpkccd cells Figure 2E

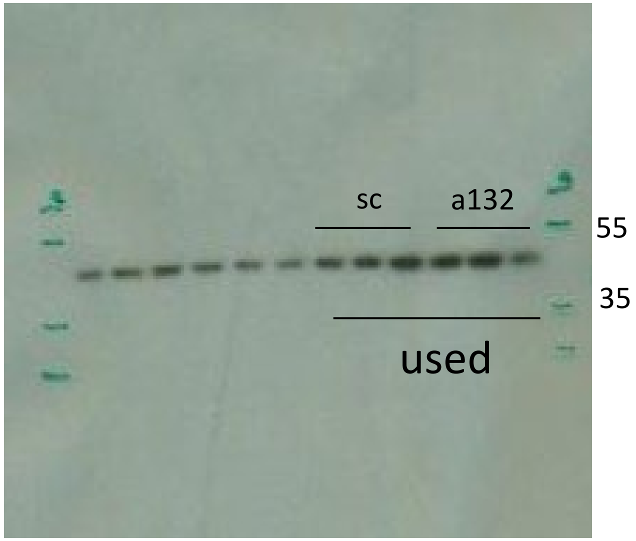

Supplementary Figure 8. Full blots (2)

Full blot p38 mmdd1 cells Figure 3A

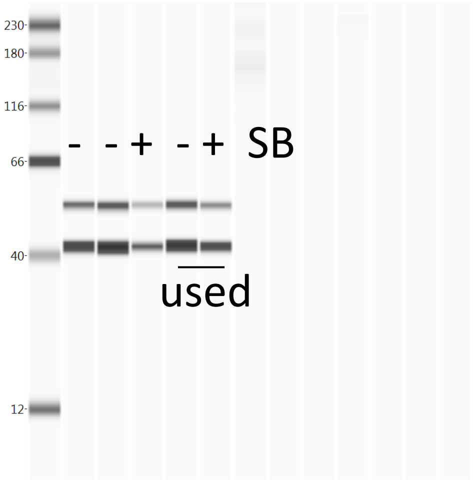

Full blot p-p38 mmdd1 cells Figure 3A

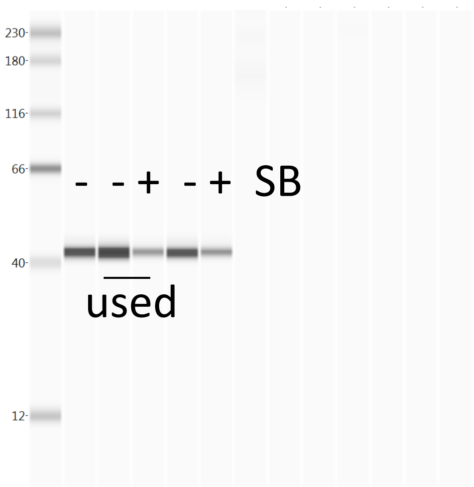

Full blot ERK mmdd1 cells Figure 3B

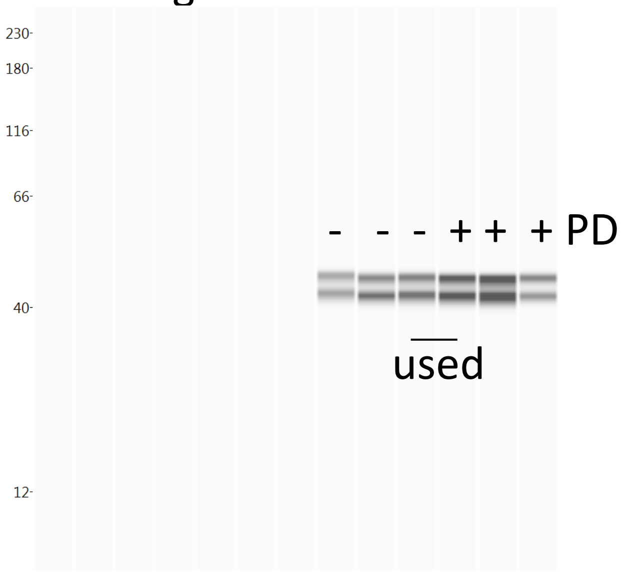

Full blot p-ERK mmdd1 Cells Figure 3B

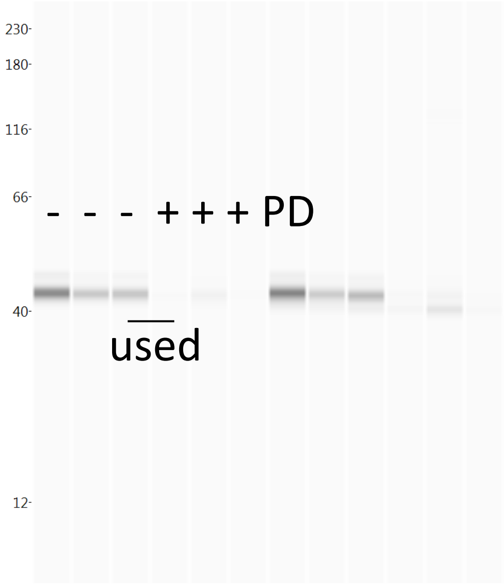

Supplementary Table 1. Physiological characteristics after miR-132 silencing. Sc = scramblemir, a132 = antagomir-132. MAP = mean arterial pressure.

**parameter**	**sc**	**a132**	**P value**
Plasma sodium (mmol/L)	150 ± 8	149 ± 9	0.80
Urine sodium (mmol/L)	189 ± 41	164 ± 36	0.24
Urine osmolality (mmol/L)	2.31 ± 0.51	1.67 ± 0.28	*0.004*
Urine output (mL)	0.59 ± 0.06	0.86 ± 0.07	0.003
Blood pressure (MAP, mmHg)	88 ± 3	79 ± 2	0.07

Supplementary Methods

Depicted are the synthetic, double-stranded oligonucleotides spanning a 60 bp region of the murine 3′ UTR of Cox2 mRNA containing the putative miR-132 binding site with or without a point mutation (in red) that was cloned downstream of the firefly luciferase reporter gene in the pMIR-report^{TM} Expression Reporter Vector System

pMIR-mCox2 sense

5'- CTAGT TAGAATTCGTGCATCAAGAA atgatgacaaataatACTGTTa
TTTATATAAATAACTAAAAA A -3'

pMIR-mCox2 antisense

5'- AGCTT TTTTTAGTTATTTATATAAA tAACAGTattatttgtcatcat TTCTTGATGCACGAATTCTA A -3'

pMIR-mCox2mut sense

5'- CTAGT TAGAATTCGTGCATCAAGAA atgatgacaaataatAGTGTTa
TTTATATAAATAACTAAAAA A
-3'

pMIR-mCox2mut antisense

5'- AGCTT TTTTTAGTTATTTATATAAA tAACACTattatttgtcatcat TTCTTGATGCACGAATTCTA A -3'
