# Supplementary material for: MicroRNA-132 regulates salt-dependent steady-state renin levels in mice
Source: Commun Biol. 2020 May 14;3:238. doi: 10.1038/s42003-020-0967-4 (PMC7224281; doi:10.1038/s42003-020-0967-4)
Supplement: Supplementary file 4 — Reporting Summary [file 42003_2020_967_MOESM4_ESM.pdf]

nature research

Corresponding author(s): Roel Bijkerk

Last updated by author(s): Apr 14, 2020

Reporting Summary

Nature Research wishes to improve the reproducibility of the work that we publish. This form provides structure for consistency and transparency in reporting. For further information on Nature Research policies, see Authors & Referees and the Editorial Policy Checklist .

Statistics

For all statistical analyses, confirm that the following items are present in the figure legend, table legend, main text, or Methods section.

n/a	Confirmed	
☐	☒	The exact sample size (*n*) for each experimental group/condition, given as a discrete number and unit of measurement
☐	☒	A statement on whether measurements were taken from distinct samples or whether the same sample was measured repeatedly
☐	☒	The statistical test(s) used AND whether they are one- or two-sided<br>*Only common tests should be described solely by name; describe more complex techniques in the Methods section.*
☒	☐	A description of all covariates tested
☐	☒	A description of any assumptions or corrections, such as tests of normality and adjustment for multiple comparisons
☐	☒	A full description of the statistical parameters including central tendency (e.g. means) or other basic estimates (e.g. regression coefficient) AND variation (e.g. standard deviation) or associated estimates of uncertainty (e.g. confidence intervals)
☒	☐	For null hypothesis testing, the test statistic (e.g. *F*, *t*, *r*) with confidence intervals, effect sizes, degrees of freedom and *P* value noted<br>*Give P values as exact values whenever suitable.*
☒	☐	For Bayesian analysis, information on the choice of priors and Markov chain Monte Carlo settings
☒	☐	For hierarchical and complex designs, identification of the appropriate level for tests and full reporting of outcomes
☒	☐	Estimates of effect sizes (e.g. Cohen's *d*, Pearson's *r*), indicating how they were calculated

Our web collection on statistics for biologists contains articles on many of the points above.

Software and code

Policy information about availability of computer code

Data collection	Compass for SW software (Protein Simple), for capillary western blot.
Data analysis	GraphPad, Qiagen Ingenuity Pathway Analysis, SPSS v25

For manuscripts utilizing custom algorithms or software that are central to the research but not yet described in published literature, software must be made available to editors/reviewers. We strongly encourage code deposition in a community repository (e.g. GitHub). See the Nature Research guidelines for submitting code & software for further information.

Data

Policy information about availability of data

All manuscripts must include a data availability statement. This statement should provide the following information, where applicable:

- Accession codes, unique identifiers, or web links for publicly available datasets
- A list of figures that have associated raw data
- A description of any restrictions on data availability

The authors declare that the main data supporting the findings of this study are available within the article and its Supplementary Information files. Extra data are available from the corresponding author upon request.

Field-specific reporting

Please select the one below that is the best fit for your research. If you are not sure, read the appropriate sections before making your selection.

☒ Life sciences ☐ Behavioural & social sciences ☐ Ecological, evolutionary & environmental sciences

For a reference copy of the document with all sections, see nature.com/documents/nr-reporting-summary-flat.pdf

Life sciences study design

All studies must disclose on these points even when the disclosure is negative.

Sample size	No power calculation was done for sample size; instead sample sizes were estimated based on previous experience with comparable experiments and tissues, plasma and urine were analyzed based on sample availability.
Data exclusions	No data was excluded. In Figure 4 and 5 not always enough urine/plasma was available to analyze the complete n of the group.
Replication	All experiments were at least repeated 3 times, and indicated in the methods and figures.
Randomization	For animal studies, groups were balanced and randomized based on body weight.
Blinding	Investigators were blinded to group allocation during data analysis.

Reporting for specific materials, systems and methods

We require information from authors about some types of materials, experimental systems and methods used in many studies. Here, indicate whether each material, system or method listed is relevant to your study. If you are not sure if a list item applies to your research, read the appropriate section before selecting a response.

Materials & experimental systems

n/a	Involved in the study	
☐	☒	Antibodies
☐	☒	Eukaryotic cell lines
☒	☐	Palaeontology
☐	☒	Animals and other organisms
☒	☐	Human research participants
☒	☐	Clinical data

Methods

n/a	Involved in the study	
☒	☐	ChIP-seq
☒	☐	Flow cytometry
☒	☐	MRI-based neuroimaging

Antibodies

Antibodies used	B-actin (Millipore; MABT825), COX-2 (Abcam; ab15191), goat-anti-rabbit-HRP (Dako; P044801-2), p38 (Cell Signaling Technology; 9212), phospho-p38 (Cell Signaling Technology; 9211s), ERK1/2 (Cell Signaling Technology; 9102s), phospho-ERK1/2 (Cell Signaling Technology; 9101S), GAPDH (Cell Signaling Technology; 5174S), NKCC2 (LSBio; LS-C313275-100), anti-rabbit-alexa568 (Molecular Probes; A-11011)
Validation	All commercial antibodies were purchased from vendors that included validation statements on the manufacturers website and were positively reviewed and cited for the correct applications. B-actin antibody was validated by manufacturer for western blot. Cell Siganling antibodies p38, p-p38, ERK1/2 and p-ERK1/2 were succesfully used and cited many times as indicated on the website of the manufacturer. COX-2 antibody was used in 165 references (as shown on abcam website) and positively reviewed 35 times. NKCC2 antibody was cited in the website of the manufacturer for a staining in a comparable setting. Secondary antodies were validated by manufacturers and succesfully used in many previous experiments in our lab. Also, for western blot, bands were at expected molecular weights.

Eukaryotic cell lines

Policy information about cell lines

Cell line source(s)	NIH3T3 and mIMCD3 cell lines were obtained from American Type Tissue Collection. MMDD-1 cell line (Dr. Bachmann Lab, Charité-Universitätsmedizin Berlin, Germany)
Authentication	NIH3T3 and mIMCD3 cell lines were authenticated using standard American Type Tissue Collection methods. MMDD-1 cell authentication was done by checking for expression of previously described genes and we confirmed the response to salt treatment of these cells.
Mycoplasma contamination	Cells were tested negatively for mycoplasma contamination.
Commonly misidentified lines (See ICLAC register)	No commonly misidentified cell lines were used.

Animals and other organisms

Policy information about studies involving animals; ARRIVE guidelines recommended for reporting animal research

Laboratory animals	Male balb/c mice, 8-9 weeks old were used.
Wild animals	the study did not involve wild animals
Field-collected samples	The study did not involve field-collected samples
Ethics oversight	The animal welfare committee of the Leiden University Medical Center approved all animal experiments and protocols.

Note that full information on the approval of the study protocol must also be provided in the manuscript.
