# Supplementary material for: MicroRNA-132 regulates salt-dependent steady-state renin levels in mice
Source: Commun Biol. 2020 May 14;3:238. doi: 10.1038/s42003-020-0967-4 (PMC7224281; doi:10.1038/s42003-020-0967-4)
Supplement: Supplementary file 3 — Peer Review File [file 42003_2020_967_MOESM3_ESM.pdf]

Reviewers' comments:

Reviewer #1 (Remarks to the Author):

The present study examined the role and mechanisms of miR132 in the macula densa cells in regulation of renin release. Authors found that miR-132 was strongly expressed in the macula densa and altered by different salt intake. Silencing miR132 increased plasma renin levels, accompanied by increased macula densa COX-2 expression and elevated PGE2. Then a series experiments in vitro were performed to examine the mechanisms for mir132 in the macula densa in control of renin release. Authors demonstrated that miR-132 targeted Cox-2 and affect subsequent PGE2 synthesis affected by low salt and high salt treatment, in a p38- and ERK1/2-independent and CREB- and salt inducible kinase (SIK)-dependent manner. Authors concluded that macula densa miR-132 plays an essential posttranscriptional regulatory role for salt-dependent in fine-tuning the synthesis and release of renin through COX-2 mediated PGE2 synthesis.
While the hypothesis is interesting and important, experimental design is pretty premature and additional data are needed to support their conclusions.

1. Authors stated that "By far the most prominent expression of miR-132 was observed in the distal tubular epithelial cells neighboring glomeruli, most likely being the cells of the macula densa." Since the role of miR-132 in the macula densa is the major objective for the present study, authors need to confirm that the observed tubular cells that express miR-132 are the macula densa cells. Immunofluorescence with double staining of NOS1 or NKCC2 is preferred.
2. Silencing miR-132 increased plasma renin concentration in vivo. While these data are intriguing and authors assume that the effects were via the macula densa, there are several questions that need to be clarified: a) did the antagomir-132 have any effect on blood pressure? b) other tubular cells are also express high level of miR-132. It is not clear if silencing miR-132 in these tubular cells have any effect on sodium reabsorption, which may affect Na delivery to the macula densa or sodium excretion that alters renin release; and c) it seems that miR-132 is expressed in glomerular capillaries. It is not clear if miR-132 exists in the afferent arterioles including JG cells. If yes, what is the role of miR-132 in regulation of vascular tone and renin activity? What is the effect of silencing miR-132 in vasculature on renin release?
3. Similar concerns exist for COX-2 inhibition as the miR-132 silencing. It seems that the COX-2 inhibitor was given systemically. It is not clear how to determine the effect was mediated by the macula densa cells.

Reviewer #2 (Remarks to the Author):

Summary

The article 'Feedback modulation of renin synthesis by macula densa microRNA-132' was authored by Zonneveld et al. The authors have hypothesized that in the mouse, miR-132 has a role in responding to low vs high salt levels. Reduction of miR-132 results in changes of expression of two downstream targets (COX-2 and PGE2), which ultimately increased renin production. This manuscript mixes in vitro cell models with a mouse animal model where the salt content of the diet was varied to support the in vitro model. The implications from this work is that miR-132 can play a role in regulation of COX-2/PGE2 to influence renin levels in response to salt intake.

Overall Impression

The experiments appear to be well-designed, described, and support the claims within the manuscript. The approach establishes the model with in vitro work and then uses animal models to test specific hypothesis. Reviewer comments below are generally focused on improving the readability of the document. However, I ask the Authors to pay specific attention to comments around statistical methods because it appears 3 different methods were used in this manuscript, which attracts negative attention. I understand that the normal distribution of data was used to

select the method of analysis, but the question this brings up is would data be significant if a student t-test was conducted instead of a MW test?

One additional consideration to bring to the Authors/Editor's attention is writing support is needed to support the clarity within this manuscript. There are run-on sentences, and sections (e.g., the Introduction) could use additional paragraphs. Additionally, throughout the Results section there is a frequent switch between present/past tenses.

Specific comments

Major comments

1. In the histology image shown in Figure 1A, the authors describe the image as 'most likely the macula dense.' Given that this is the first data point in the manuscript, and at some level the entire hypothesis requires miR-132 to be expressed in the macula densa, can the authors perform additional work to confirm the nature of these cells? For example, providing images to a veterinary pathologist for confirmation? Or take serial sections of the kidney and stain 1 for miR-132 and the next slice for a histological marker? Related to Figure 1A, If the Authors are not confident in identifying the macula dense, how are they confident in the microdissection of Figure 5F (or in Supplementary Figure 6)?

2. Its not clear in the figures, why Standard Deviation was used for some error bars and why Interquartile Range was used for others. For example, Figure 2G used IR while Figure 2H uses SD, even through they are the same general type of experiment. Please explain the preference for IR vs. SD. Would the use of Standard Error for all experiments be more appropriate?

In the methods section, the statistical analyses are stated as either a student's t-test or a Mann-Whithey t-test 'when appropriate.' How was one test decided vs. another? If there was a specific type of experiment that is commonly analyzed with a M-W t-test, please state as much.

Additionally, the use of linear modelling in 'SPSS with Sidak post-hoc test' is admittedly outside of my area of familiarity. However, it's confusing why this test was applied to Figure 4G (urinary PGE2 levels) but not the similar assessment of plasma renin levels in Figure 4H.

The unfortunate ambiguity that these different statistical methods & errors bars creates is one where the significance of the data depends on the method used.

3. When measuring analytes in urine samples typically urinary creatinine is used to normalize against volume. This is true for both analysis of protein and miR. The Methods section simply states that the kits were used following manufacture's instructions. Please state the normalization method in the text (figure legend or Methods section).

This is especially important given a comment in the Results section on page 7 for Figure 4F (…celexocib treatment did not change urine output…which excludes indirect effects via volume changes) because the implication is that nothing was used to for urine normalization.

This creates a concern around Figure 5C, which is a measure of urinary PGE2 levels from low/normal/high salt diets. The stated urinary outputs in the table of Figure 5B shows that high salt increases water intake and urine output. So was the decreased levels of PGE2 a result of a diluted sample? Or did the levels actually drop? Its unclear if the Authors used the urine volume as a surrogate for normalization to PGE2.

It the Authors have remaining urinary samples, please re-analyze them for something like creatinine to assist in normalization. If the samples are not available, then I would recommend statements in the text that the values were not normalized. Additionally, please ensure that all measured urine volumes are reported in the Supplementary Information, which can easily be linked to an experiment. For example, it looks like Supplementary Table 1 refers to the data in

Figure 1, but then were is the respective table for Figure 4?

Minor comments

1. Related to the use of tenses, the more common style preference is to describe work in the past. For example, when describing an experiment instead of writing 'we treated the cells,' consider stating 'the cells were treated.'
2. Figure 1B can be described with text and is not necessary as a figure. If removed, please make the graphs in Fig 1C and 1D larger to fill the space.
3. The Figure legends often describe data/results/conclusions and not they key features of an experiment that would help a reader reach their own interpretation. For example, the legend for Figure gives CV values and a conclusion in the legend.
4. For Figures 2G and 2H, both graphs are composites of separate assays. For clarity, I'd recommend breaking 2G into two side-to-side figures, and the same for 2H.
5. For Figure, 2K, please include a description of the Control (ctrl) treatment in the figure legend.
6. Figure 6 legend, 'sodiumchloride' is a typo. This is otherwise a very nice figure.
7. In Results section (page 10) the second paragraph states 'we validated…' I would change the word 'validated' to 'demonstrated.' Likewise comment for the used of 'validated' on pages 4 and 5.
8. In Figure 5, there are multiple graphs (C, D, E, G and H) that display the P-value for statistically not-significant data sets (e.g., $P > 0.05$). I recommend either removing the P-values, or adding a 'n.s.' on top of the P-value to clearly indicate that the data is not statistically significant. Please do this for other experiments where the Authors provide P values that are >0.05.
9. The last paragraph in the Discussion section (page 12) takes a leap of logic relating the findings of the paper to human physiology (e.g., implication on human diet). Please revise the paragraph to focus on the data shown in the paper, which was for a mouse model.
10. In Supplementary Figure 1, the Figure legend describes 'preliminary' data. Why is this preliminary and what is needed for this data to have confidence behind it?
11. In the Reference Section, citations 12, 25, and 30 appear to be in the wrong format. Please resolve and double check the other citations.

Reviewer #3 (Remarks to the Author):

In this manuscript van Zonneveld et al pose an interesting hypothesis, that miRNA-132 plays a role in the regulation of renin synthesis/release via a direct inhibition of Cox-2 at the 3'UTR site. The authors present promising in vitro and in vivo data, however further experiments will be necessary to confirm or reject their hypothesis.

Concerns:

1) Whereas miRNA-132 is highly expressed in the macula densa cells in response to low salt, it is also expressed in other cell types such as renin cells, interstitial cells, glomerular cells and additional tubular cells. Therefore, it is necessary to delete miRNA-132 exclusively in macula densa cells, otherwise, the effects observed using antagomirs could be systemic through other cells/pathways. Furthermore, a more precise characterization of the expression pattern of miRNA-132 is needed (Figure 1A shows expression also in an intrarenal artery)
2) The increase in plasma renin as a result of intravenous injection of antagomir-132 (Figure1) is not convincing. The normal wide range of plasmatic renin in control mice (from ~5000-17500 pg/ml), shows only one mouse out of eight with a slightly higher value of plasma renin. This also invalidates the results shown in Figures 4 and 5. A higher number of mice are needed.
3) Luciferase assays: To demonstrate specificity, need to do the same luciferase assays mutating only the bases complementary to the miRNA sequence, mutating the entire miRNA targeting site (e.g., using reversed strand), or deleting the seed region of miRNA-132 and adding mimic miRNA-132. Otherwise the effect could be due to other targets of 132 in other genes. It is not enough to do experiments using scramble miRNAs.
4) In vitro studies need more detailed methods, how many cells /wells were used per experiment? How many technical replicates? Three individual values with no further information, just a

statement of having each experiment repeated three times, is not enough.
5) The statement that animals given “low salt diet in combination with antagomir-132 displayed a further increase in PGE2 and plasma renin levels” (Figure 5H and I) is based on a “trend” ($p=0.06$) for PGE2 and 4 animals with plasma renin values BELOW one animal with just low sodium diet. As mentioned in 2) the n= needs to be increased.
6) In the discussion please explain how an inhibitor of Cox2/renin play a meaningful role in homeostasis, when both increase despite the increase of the inhibitor. Just “modulation” is not enough. In other words, how do you envision that this system works?
7) The statement in the discussion regarding a potential role of miRNA-132 in the recruitment of CD44 is not substantiated by the data presented and should be eliminated. Particularly since the CD44 data is questionable at best (reference 41)
8) What are the units for Figure 2G right side?
9) Supplementary Figure 7: Please remove from the legend the abbreviations. They do not correspond to this figure.

Rebuttal for manuscript: "Feedback modulation of renin synthesis by macula densa microRNA-132" (Manuscript ID COMMSBIO-19-1743-T).

Following examination by expert outside Reviewers and the Editors, we were provided the opportunity to submit a revised version of our manuscript pending our ability to address the specified points. We appreciate the constructive criticism provided by the reviewers and believe it has helped us to strongly improve our manuscript.

Below we provide our detailed responses to the Reviewers concerns.

Reviewer #1 (Remarks to the Author):

The present study examined the role and mechanisms of miR132 in the macula densa cells in regulation of renin release. Authors found that miR-132 was strongly expressed in the macula densa and altered by different salt intake. Silencing miR132 increased plasma renin levels, accompanied by increased macula densa COX-2 expression and elevated PGE2. Then a series experiments in vitro were performed to examine the mechanisms for mir132 in the macula densa in control of renin release. Authors demonstrated that miR-132 targeted Cox-2 and affect subsequent PGE2 synthesis affected by low salt and high salt treatment, in a p38- and ERK1/2-independent and CREB- and salt inducible kinase (SIK)-dependent manner. Authors concluded that macula densa miR-132 plays an essential posttranscriptional regulatory role for salt-dependent in fine-tuning the synthesis and release of renin through COX-2 mediated PGE2 synthesis.
While the hypothesis is interesting and important, experimental design is pretty premature and additional data are needed to support their conclusions.

1. Authors stated that "By far the most prominent expression of miR-132 was observed in the distal tubular epithelial cells neighboring glomeruli, most likely being the cells of the macula densa." Since the role of miR-132 in the macula densa is the major objective for the present study, authors need to confirm that the observed tubular cells that express miR-132 are the macula densa cells. Immunofluorescence with double staining of NOS1 or NKCC2 is preferred.

Answer: Although we are convinced these cells are the macula densa cells, we wanted to carefully choose our words and chose to state 'most likely', which is arguably not the best choice of words. Nonetheless, we agree it is important to provide additional evidence showing that these cells are indeed macula densa cells. We considered co-staining with the markers as suggested by the reviewer but felt it would still be inconclusive as these markers are not specific for only macula densa cells. Therefore, as also suggested by another reviewer, we consulted a renal pathologist (Dr. Ingeborg Bajema, she is an internationally recognized renal pathologist at the Leiden University Medical Center) and asked her, based on the in situ hybridization, what cell type showed this intense staining, and she also indicated these are macula densa cells. We included this analysis in the text of the results section:

'By far the most prominent expression of miR-132 was observed in the distal tubular epithelial cells neighboring glomeruli, being the cells of the macula densa (Figure 1A), of which the identity was confirmed by a renal pathologist'

2. Silencing miR-132 increased plasma renin concentration in vivo. While these data are intriguing and authors assume that the effects were via the macula densa, there are several questions that need to be clarified: a) did the antagomir-132 have any effect on blood pressure? b) other tubular

cells are also express high level of miR-132. It is not clear if silencing miR-132 in these tubular cells have any effect on sodium reabsorption, which may affect Na delivery to the macula densa or sodium excretion that alters renin release; and c) it seems that miR-132 is expressed in glomerular capillaries. It is not clear if miR-132 exists in the afferent arterioles including JG cells. If yes, what is the role of miR-132 in regulation of vascular tone and renin activity? What is the effect of silencing miR-132 in vasculature on renin release?

Answer: To summarize these set of remarks, we think the reviewer mainly indicates that it is not possible, based on our experimental setup and data, to conclude that the effects of silencing miR-132 on renin levels in mediated via the macula densa. We have been considering these remarks and although we are convinced that our observed effects are mediated (at least partly) via the macula densa, the reviewer is right that we cannot be 100% sure of this, given our systemic silencing of miR-132, as well as Celecoxib administration. The only way to be 100% sure would be to employ approaches such as conditional knockout models or localized administration, which are either virtually impossible and/or would not be possible within a reasonable time and effort. Nonetheless, I believe that the main message of the manuscript, 'feedback modulation of renin by miR-132' still holds, we just cannot be absolutely sure that this is mediated directly via the macula densa. As such, we have adjusted the manuscript to where the focus lies just on this main message (the title now has become "Feedback modulation of renin synthesis by microRNA-132"), while a role of the macula densa is only suggested. In retrospect, we may have drawn too bold conclusions on the direct macula densa effect, but believe that in this slightly differently written version would be just as interesting for the reader!!!

In addition, more specifically answering the remarks, silencing miR-132 indeed slightly reduces blood pressure (P=0.07) which is indicated in supplementary Table 1. However, this is a result of the diuretic effect, as previously described (Bijkerk et al, 2018, Am J Physiol Ren Physiol), which is why we performed control experiments with ddAVP administration (Figure 4G-H), where we could investigate the role of miR-132 in renin synthesis without the diuretic effects.

Furthermore, the proposed alternative ways of action of miR-132 could indeed very well be relevant for the observed phenotype and have therefore been discussed in the discussion section:

"Although our data describe a macula densa-centred mechanism, miR-132 is strongly expressed in other cell types as well, including proximal tubular epithelial cells, collecting duct (that also express COX-2 and renin) and vascular cells. Given our systemic silencing of miR-132, this suggests possible involvement through these cell types as well. The same applies to the systemic use of Celecoxib for COX-2 inhibition, which may affect multiple cell types. For example, we previously found miR-132 inhibition to affect NHE3 (sodium–hydrogen exchanger 3) expression[12], which could result in an effect on sodium reabsorption, which subsequently may affect sodium delivery to the macula densa or sodium excretion that alters renin release. Furthermore, a role for miR-132 in arterial cells has been previously described in relation to hypertension[24], which may translate to altered signaling to the JGA impacting renin release. As such, it may very well be possible that macula densa- and JGA-independent effects contribute to the in vivo miR-132 inhibition-induced increase in COX-2/PGE2 and renin. Nonetheless, given that COX-2 was increased in macula densas as a result of miR-132 silencing, while also our salt diet experiments indicate physiological relevance of miR-132 in the macula densa, our data suggest at least a partial direct macula densa and JGA mediated effect."

3. Similar concerns exist for COX-2 inhibition as the miR-132 silencing. It seems that the COX-2 inhibitor was given systemically. It is not clear how to determine the effect was mediated by the macula densa cells.

Answer: We agree as discussed above and have changed the manuscript and conclusions accordingly.

Reviewer #2 (Remarks to the Author):

Summary

The article 'Feedback modulation of renin synthesis by macula densa microRNA-132' was authored by Zonneveld et al. The authors have hypothesized that in the mouse, miR-132 has a role in responding to low vs high salt levels. Reduction of miR-132 results in changes of expression of two downstream targets (COX-2 and PGE2), which ultimately increased renin production. This manuscript mixes in vitro cell models with a mouse animal model where the salt content of the diet was varied to support the in vitro model. The implications from this work is that miR-132 can play a role in regulation of COX-2/PGE2 to influence renin levels in response to salt intake.

Overall Impression

The experiments appear to be well-designed, described, and support the claims within the manuscript. The approach establishes the model with in vitro work and then uses animal models to test specific hypothesis. Reviewer comments below are generally focused on improving the readability of the document. However, I ask the Authors to pay specific attention to comments around statistical methods because it appears 3 different methods were used in this manuscript, which attracts negative attention. I understand that the normal distribution of data was used to select the method of analysis, but the question this brings up is would data be significant if a student t-test was conducted instead of a MW test?

Answer: We thank the reviewer for his positive review of the manuscript. With regard to the statistical tests, we understand that the use of different statistical tests is confusing, but we have consulted a statistician that advised us in choosing the most appropriate statistical test for each experiment. In fact, using simple straightforward statistical tests everywhere (e.g. t-tests) would on average have actually lowered our p-values and thus increased statistical significance. Further specific answers regarding statistical tests are given below.

One additional consideration to bring to the Authors/Editor's attention is writing support is needed to support the clarity within this manuscript. There are run-on sentences, and sections (e.g., the Introduction) could use additional paragraphs. Additionally, throughout the Results section there is a frequent switch between present/past tenses.

Answer: We have carefully evaluated our manuscript and improved these issues where necessary. Furthermore, we asked a native speaker to 'double check' our manuscript to improve readability and have incorporated his suggestions.

Specific comments

Major comments

1. In the histology image shown in Figure 1A, the authors describe the image as 'most likely the macula dense.' Given that this is the first data point in the manuscript, and at some level the entire hypothesis requires miR-132 to be expressed in the macula densa, can the authors perform additional work to confirm the nature of these cells? For example, providing images to a veterinary pathologist for confirmation? Or take serial sections of the kidney and stain 1 for miR-132 and the next slice for a histological marker? Related to Figure 1A, If the Authors are not confident in identifying the macula dense, how are they confident in the microdissection of Figure 5F (or in Supplementary Figure 6)?

Answer: Although we are convinced these cells are the macula densa cells, this was a subjective call, and we wanted to carefully choose our words and chose to state 'most likely', which is arguably not the best choice of words. Nonetheless, we agree it is important to provide additional evidence showing that these cells are indeed macula densa cells. Therefore, we followed your excellent suggestion and consulted a renal pathologist (Dr. Ingeborg Bajema, she is an internationally recognized renal pathologist at the Leiden University Medical Center) and asked her, based on the in situ hybridization, what cell type showed this intense staining. She also indicated these are macula densa cells. We included this analysis in the text of the results section:

'By far the most prominent expression of miR-132 was observed in the distal tubular epithelial cells neighboring glomeruli, being the cells of the macula densa (Figure 1A), of which the identity was confirmed by a renal pathologist'

2. Its not clear in the figures, why Standard Deviation was used for some error bars and why Interquartile Range was used for others. For example, Figure 2G used IR while Figure 2H uses SD, even through they are the same general type of experiment. Please explain the preference for IR vs. SD. Would the use of Standard Error for all experiments be more appropriate?

Answer: We agree that it would appear more consistent to use the same indication of error, but these aspects were also brought to our attention by a statistician. He advised us how to plot the data and indicated that when the distribution of the results could not be considered as normal, they should be presented as median and interquartile (IQ1-IQ3) instead of mean and SD. As such, we have followed his advice.

In the methods section, the statistical analyses are stated as either a student's t-test or a Mann-Whithey t-test 'when appropriate.' How was one test decided vs. another? If there was a specific type of experiment that is commonly analyzed with a M-W t-test, please state as much.

Answer: When two groups were compared, we first assessed if the data was distributed normally. If so, we chose student's t-test, if not we chose the Mann-Whitney test. This has now been explicitly stated in the methods section. We hope this way it is clear for the reader as to why and when specific statistical tests were used.

Additionally, the use of linear modelling in 'SPSS with Sidak post-hoc test' is admittedly outside of my area of familiarity. However, it's confusing why this test was applied to Figure 4G (urinary PGE2 levels) but not the similar assessment of plasma renin levels in Figure 4H.

The unfortunate ambiguity that these different statistical methods & errors bars creates is one where the significance of the data depends on the method used.

Answer: Since figure 4F involves more than two groups (like Figure 4G), a different statistical test has to be used for comparing the groups. In this case this should be an ANOVA-like statistical test. However, since the ANOVA test cannot compare groups with different group sizes, we had to employ SPSS, where linear modelling is a variant of the ANOVA but can deal with different group sizes (and where a Sidak post-hoc test is apparently a common test to obtain p-values).

Taken together, we completely understand that these different statistical tests may appear ambiguous and appear to provide different statistical outcomes, but in fact we have done the utmost to use the most appropriate test in each situation, as suggested by a statistician. We hope the reviewer can appreciate the effort we put in the statistical analyses (even though we understand this makes the interpretation slightly more difficult).

3. When measuring analytes in urine samples typically urinary creatinine is used to normalize against volume. This is true for both analysis of protein and miR. The Methods section simply states that the kits were used following manufacture's instructions. Please state the normalization method in the text (figure legend or Methods section).

This is especially important given a comment in the Results section on page 7 for Figure 4F (… celexocib treatment did not change urine output…which excludes indirect effects via volume changes) because the implication is that nothing was used to for urine normalization.

This creates a concern around Figure 5C, which is a measure of urinary PGE2 levels from low/normal/high salt diets. The stated urinary outputs in the table of Figure 5B shows that high salt increases water intake and urine output. So was the decreased levels of PGE2 a result of a diluted sample? Or did the levels actually drop? Its unclear if the Authors used the urine volume as a surrogate for normalization to PGE2.

It the Authors have remaining urinary samples, please re-analyze them for something like creatinine to assist in normalization. If the samples are not available, then I would recommend statements in the text that the values were not normalized.

Answer: We have performed all measurements on 24 hr urine and normalized to urine volumes in all PGE2 measurements, except indeed in figure 5C. We now clearly indicated this in the methods section. We presented Figure 5C as concentration (we also noticed a slight error in scale, with no effect on results, but which is now adjusted) since we observed this extreme amount of urine in the high salt group which makes it difficult to interpret the total amount of PGE2. Therefore we chose to present this one as pg/mL and also indicate the urine volumes in figure 5B. We hope the reviewer can follow this line of reasoning and agrees this is the best way to present the data.

Nonetheless, in retrospect, we agree we should have analysed creatinine values for the urines, but unfortunately we now only had enough urine left from the experiment described in figure 4C.To explore the effects of creatinine normalization at least for that experiment, we measured creatinine

in these samples and plotted the data as ratios to creatinine, as exemplified below (mean urine creatinine (± SD) in sc group = 57 ± 7 mg/dL and 50 ± 7 mg/dL in a132 group):

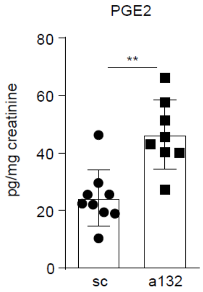

Alternative Figure 4C. PGE2 levels represented per mg creatinine. **P<0.01. Data are represented as median ± interquartile range (IQ1-IQ3)

This does not alter the results and outcome and even though statistical significance improved here, we prefer to present our data as per 24 hour urine similar as in the clinic as we feel this is most representative of 'absolute quantities' in our experiments. In addition, as also indicated above, we unfortunately do not have urines available for the other experiments. Following the advice of the reviewer, we have stated in the text that values were normalized to urine volume and not normalized to creatinine.

Minor comments

1. Related to the use of tenses, the more common style preference is to describe work in the past. For example, when describing an experiment instead of writing 'we treated the cells,' consider stating 'the cells were treated.'

Answer: We have now implemented this style of writing throughout the manuscript.

2. Figure 1B can be described with text and is not necessary as a figure. If removed, please make the graphs in Fig 1C and 1D larger to fill the space.

Answer: Although we agree figure 1B is not essential, we believe that it provides the reader a visual guide as to which *in vivo* experiment follows in the different figures. Particularly in figures 4 and 5, where we use the same type of figures, we believe it makes it clear for the readers when data of the next *in vivo* experiment starts in a figure. As such, we prefer to also keep figure 1b, unless the reviewer feels it distracts from the main message.

3. The Figure legends often describe data/results/conclusions and not they key features of an experiment that would help a reader reach their own interpretation. For example, the legend for Figure gives CV values and a conclusion in the legend.

Answer: We have adjusted the legends and tried to include the appropriate experimental details (such as n).

4. For Figures 2G and 2H, both graphs are composites of separate assays. For clarity, I'd recommend breaking 2G into two side-to-side figures, and the same for 2H.

Answer: As suggested, we have split these graphs, which are now presented as Figures 2G, 2H, 2I and 2J.

5. For Figure, 2K, please include a description of the Control (ctrl) treatment in the figure legend.

Answer: It has now been included that the control treatment is scramblemir treated cells.

6. Figure 6 legend, 'sodiumchloride' is a typo. This is otherwise a very nice figure.

Answer: This has been corrected, thank you.

7. In Results section (page 10) the second paragraph states 'we validated…' I would change the word 'validated' to 'demonstrated.' Likewise comment for the used of 'validated' on pages 4 and 5.

Answer: we agree this is a better word choice and have adjusted this.

8. In Figure 5, there are multiple graphs (C, D, E, G and H) that display the P-value for statistically not-significant data sets (e.g., P > 0.05). I recommend either removing the P-values, or adding a 'n.s.' on top of the P-value to clearly indicate that the data is not statistically significant. Please do this for other experiments where the Authors provide P values that are >0.05.

Answer: Personally, I don't think there is much difference between P=0.04 and P=0.06 as P=0.05 is an arbitrarily chosen cut-off point for statistical significance. Sometimes I am truly convinced a difference with p-value of 0.06 is really true, while sometimes I see p-values of 0.01 which I do not believe. As such, I prefer to leave it up to the reader to interpret the value of p-values around P=0.05, which I therefore usually choose to include in the figures, as I believe it is the most objective way to present the data. Nonetheless, I understand it is common to not include these values. For now I have chosen to keep the values in the graphs, but if the reviewer feels this is not ok, I will remove the p-values from the graphs.

9. The last paragraph in the Discussion section (page 12) takes a leap of logic relating the findings of the paper to human physiology (e.g., implication on human diet). Please revise the paragraph to focus on the data shown in the paper, which was for a mouse model.

Answer: Agreed, in retrospect this has been too bold of a statement, which we therefore completely removed.

10. In Supplementary Figure 1, the Figure legend describes 'preliminary' data. Why is this preliminary and what is needed for this data to have confidence behind it?

Answer: This was a bad choice of words, as we agree the term preliminary indicates it does not have confidence but we obviously are confident about the data, else we of course would not have included this. Thank you for pointing this out. We have changed 'preliminary data' simply to 'profiling data'.

11. In the Reference Section, citations 12, 25, and 30 appear to be in the wrong format. Please resolve and double check the other citations.

Answer: We thank the reviewer for noticing this and have adjusted these references to the appropriate format.

Reviewer #3 (Remarks to the Author):

In this manuscript van Zonneveld et al pose an interesting hypothesis, that miRNA-132 plays a role in the regulation of renin synthesis/release via a direct inhibition of Cox-2 at the 3'UTR site. The authors present promising in vitro and in vivo data, however further experiments will be necessary to confirm or reject their hypothesis.

Concerns:

1) Whereas miRNA-132 is highly expressed in the macula densa cells in response to low salt, it is also expressed in other cell types such as renin cells, interstitial cells, glomerular cells and additional tubular cells. Therefore, it is necessary to delete miRNA-132 exclusively in macula densa cells, otherwise, the effects observed using antagomirs could be systemic through other cells/pathways. Furthermore, a more precise characterization of the expression pattern of miRNA-132 is needed (Figure 1A shows expression also in an intrarenal artery)

Answer: To summarize these set of remarks, we think the reviewer mainly indicates that it is not possible, based on our experimental setup and data, to conclude that the effects of silencing miR-132 on renin levels in mediated via the macula densa. We have been considering these remarks and although we are convinced that our observed effects are mediated (at least partly) via the macula densa, the reviewer is right that we cannot be 100% sure of this, given our systemic silencing of miR-132 and given the presence of miR-132 in many cellular compartments. The only way to be 100% sure would be to employ approaches such as conditional knockout models or localized administration, which are either virtually impossible and/or would not be possible within a reasonable time and effort. Nonetheless, I believe that the main message of the manuscript, 'feedback modulation of renin by miR-132' still holds, we just cannot be absolutely sure that this is mediated directly via the macula densa. As such, we have adjusted the manuscript to where the focus lies just on this main message (the title now has become "Feedback modulation of renin synthesis by microRNA-132"), while a role for the macula densa is only suggested. In retrospect, we may have drawn too bold conclusions on the direct macula densa effect, but believe that in this slightly differently written version would be just as interesting for the reader!

Furthermore, since the presence of miR-132 in other cells indeed suggests additional alternative ways of action of miR-132 relevant for the observed phenotype, we have discussed this in the discussion section:

"Although our data describe a macula densa-centred mechanism, miR-132 is strongly expressed in other cell types as well, including proximal tubular epithelial cells, collecting duct (that also express COX-2 and renin) and vascular cells. Given our systemic silencing of miR-132, this suggests possible involvement through these cell types as well. The same applies to the systemic use of Celecoxib for COX-2 inhibition, which may affect multiple cell types. For example, we previously found miR-132 inhibition to affect NHE3 (sodium–hydrogen exchanger 3) expression[12], which could result in an effect on sodium reabsorption, which subsequently may affect sodium delivery to the macula densa or

sodium excretion that alters renin release. Furthermore, a role for miR-132 in arterial cells has been previously described in relation to hypertension[24], which may translate to altered signaling to the JGA impacting renin release. As such, it may very well be possible that macula densa- and JGA-independent effects contribute to the in vivo miR-132 inhibition-induced increase in COX-2/PGE2 and renin. Nonetheless, given that COX-2 was increased in macula densas as a result of miR-132 silencing, while also our salt diet experiments indicate physiological relevance of miR-132 in the macula densa, our data suggest at least a partial direct macula densa and JGA mediated effect."

2) The increase in plasma renin as a result of intravenous injection of antagomir-132 (Figure1) is not convincing. The normal wide range of plasmatic renin in control mice (from ~5000-17500 pg/ml), shows only one mouse out of eight with a slightly higher value of plasma renin. This also invalidates the results shown in Figures 4 and 5. A higher number of mice are needed.

Answer: We consulted a statistician who advised us on the statistical analyses to be used for each experiment. As such, we found the statistically significant differences as presented, and we are convinced this represents true differences. Unfortunately we indeed observe quite some variation, but this makes it arguably even more impressive that we find a significant difference, in particular with tightly controlled physiological systems such as the RAAS.

Nonetheless, we agree that, for in particular figure 5, a higher number of mice would be beneficial to support the conclusions. We aimed to perform additional mice experiments, but unfortunately, due to strict legislative restrictions on animal experiments in the Netherlands, we are not allowed to do additional experiments unless we apply for a new permit, which takes 9 months on average. As such, doing new experiments would take at least a year, which would be, to our opinion, beyond a reasonable time. We do believe though that we provide ample *in vitro* and *in vivo* evidence for a regulatory role of miR-132 in renin synthesis.

3) Luciferase assays: To demonstrate specificity, need to do the same luciferase assays mutating only the bases complementary to the miRNA sequence, mutating the entire miRNA targeting site (e.g., using reversed strand), or deleting the seed region of miRNA-132 and adding mimic miRNA-132. Otherwise the effect could be due to other targets of 132 in other genes. It is not enough to do experiments using scramble miRNAs.

Answer: We agree this would be a very valuable addition and have therefore tested a construct with a mutated binding sites in the targeting site. We chose to incorporate a point mutation, which we believe would even provide stronger evidence for specific binding of miR-132 to the 3'UTR of Cox-2. As shown below, inserting this point mutation reversed the antagomir-132 mediated effect which further indicates a direct binding. The results and methods sections have been adjusted accordingly.

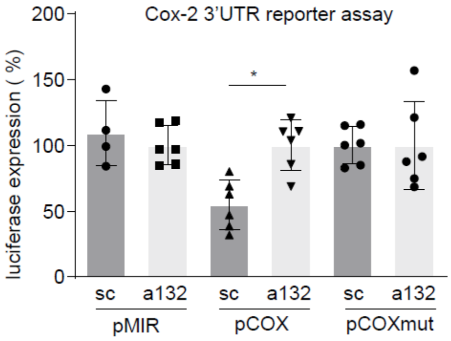

Novel Figure 2B

4) In vitro studies need more detailed methods, how many cells /wells were used per experiment? How many technical replicates? Three individual values with no further information, just a statement of having each experiment repeated three times, is not enough.

Answer: We agree this is valuable information and have now included this in the legends. Generally speaking, n=3 means 3 independent experiments in our studies, not just a technical replicate.

5) The statement that animals given "low salt diet in combination with antagomir-132 displayed a further increase in PGE2 and plasma renin levels" (Figure 5H and I) is based on a "trend" (p=0.06) for PGE2 and 4 animals with plasma renin values BELOW one animal with just low sodium diet. As mentioned in 2) the n= needs to be increased.

Answer: As indicated above in 2), we do not agree with this interpretation of data points. Nonetheless, we do agree with the fact that including more animals would provide stronger evidence for this statement. Unfortunately though, as also explained above, we did not have the ethical permission to perform these experiments within a reasonable time.

6) In the discussion please explain how an inhibitor of Cox2/renin play a meaningful role in homeostasis, when both increase despite the increase of the inhibitor. Just "modulation" is not enough. In other words, how do you envision that this system works?

Answer: The RAAS system is essential for maintaining the body's water and electrolyte balance and for blood pressure regulation. Such physiological regulatory processed need to be tightly controlled as this is crucial to proper body function. It also needs to be able to responds to changes acutely and for more prolonged periods. In this type of situation, miRNA are especially suited to provide modest feedback, as they are considered fine-tuners of biological processes. They can also simultaneously repress multiple genes to directly influence the output of functionally related biological pathways. Taken together, we envision miR-132 to be such an important fine tuner in the regulation of renin synthesis, such that the system can be balanced within subtle ranges; it is one of the factors that will keep the system from going out of control. This has now been integrated in the discussion.

7) The statement in the discussion regarding a potential role of miRNA-132 in the recruitment of CD44 is not substantiated by the data presented and should be eliminated. Particularly since the CD44 data is questionable at best (reference 41)

Answer: we agree this statement is too bold and have therefore removed it.

8) What are the units for Figure 2G right side?

Answer: To clarify this figure we chose to now split the graphs into figures 2G and 2H. Both have 'units' of relative expression.

9) Supplementary Figure 7: Please remove from the legend the abbreviations. They do not correspond to this figure.

Answer: thanks for noticing this error, this has been corrected.

Reviewers' comments:

Reviewer #1 (Remarks to the Author):

The authors are very responsive to the previous comments. Most of the concerns have been addressed. This reviewer only has 2 suggestions for the authors to consider.

1. The only way to determine macula densa cells is by double staining with NKCC2 and NOS1 (or COX2 in rodents). All other methods are inconclusive.
2. While it is possible as authors stated that "our data suggest at least a partial direct macula densa and JGA mediated effect.", however, it is still cannot exclude, nor confirm the significance of the miR-132 in other cells and tissues.

Reviewer #2 (Remarks to the Author):

No additional comments. Credit to the authors for thoroughly addressing previous major & minor comments. I appreciate that a renal pathologist was consulted, and that the choice of statistical analysis was explained.

Reviewer #3 (Remarks to the Author):

1) It is very confusing how the authors alternate between renin synthesis and renin release throughout the manuscript, starting from the title through the very end. The authors did not study renin synthesis. The did not study renin release either: what they measured is steady-state plasma renin (the result of release and clearance of renin)

2) As mentioned before, the increase in plasma renin as a result of intravenous injection of antagomir-132 (Figure1) is not convincing. The normal wide range of plasmatic renin in control mice (from ~5000-17500 pg/ml), shows only one mouse out of eight with a slightly higher value of plasma renin. This also invalidates the results shown in Figures 4 and 5. A higher number of mice is needed. This mayor concern was not addressed.

3) As mentioned before, the statement that animals given "low salt diet in combination with antagomir-132 displayed a further increase in PGE2 and plasma renin levels" (Figure 5H and I) is based on a "trend" (p=0.06) for PGE2, that is PGE2 does NOT change. Based on your own data, the logical conclusion is that the increase in renin is independent of PGE2. In any case, the "n" needs to be increased. Furthermore, in the results section, you conclude that this data supports your hypothesis that the expression of miR-132 is salt-sensitive and serves as a negative feedback regulator of COX-2/PGE2-mediated renin synthesis. Please note that you are not showing data regarding renin synthesis or release by that matter: you are measuring steady-state renin levels in plasma. I am sorry about the limitations you may face at your institution, but research requires rigor, and the data should be convincing in order to be published.

4) The sample size for the in vitro experiments is not acceptable. For example, as stated in Figure 2B, n=4-6 (independent wells) is still n=1. All the wells are "independent". You need to average all the independent wells and repeat the whole experiment at least two extra times to have an n=3.

Rebuttal for manuscript: "Feedback modulation of renin levels by microRNA-132" (Manuscript ID COMMSBIO-19-1743A).

Below we provide our detailed responses to the Reviewers concerns.

Reviewer #1 (Remarks to the Author):

The authors are very responsive to the previous comments. Most of the concerns have been addressed. This reviewer only has 2 suggestions for the authors to consider.

1. The only way to determine macula densa cells is by double staining with NKCC2 and NOS1 (or COX2 in rodents). All other methods are inconclusive.

Answer: We fully agree that double staining with NKCC2, NOS1 or COX2 would provide additional evidence for the macula densa identity of the cells (as also illustrated in Supplementary Figure 6). However, also these markers are not 100% specific and would leave some uncertainty. As such, the typical morphology of macula densa cells, as well as the confirmation by a renal pathologist would in our opinion make it very likely (and we are absolutely convinced) that the cells are indeed macula densa cells. Nonetheless, we have changed the text such that it does not claim 100% certainty but describes that these are most likely cells of the macula densa.

2. While it is possible as authors stated that "our data suggest at least a partial direct macula densa and JGA mediated effect.", however, it is still cannot exclude, nor confirm the significance of the miR-132 in other cells and tissues.

Answer: We agree and in the discussion we have tried to carefully describe that it is possible that other cells and tissue may also be involved, while dedicated studies would be necessary to determine this.

Reviewer #2 (Remarks to the Author):

No additional comments. Credit to the authors for thoroughly addressing previous major & minor comments. I appreciate that a renal pathologist was consulted, and that the choice of statistical analysis was explained.

Answer: we thank the reviewer for the positive review.

Reviewer #3 (Remarks to the Author):

1) It is very confusing how the authors alternate between renin synthesis and renin release throughout the manuscript, starting from the title through the very end. The authors did not study renin synthesis. The did not study renin release either: what they measured is steady-state plasma renin (the result of release and clearance of renin)

Answer: This is a good point raised by the reviewer. We agree that we cannot distinguish between renin synthesis and release and have therefore changed the text throughout our manuscript to describe a change in (steady-state) plasma levels. E.g. the title has now been changed to *"Feedback modulation of renin levels by microRNA-132".*

2) As mentioned before, the increase in plasma renin as a result of intravenous injection of antagomir-132 (Figure1) is not convincing. The normal wide range of plasmatic renin in control mice (from ~5000-17500 pg/ml), shows only one mouse out of eight with a slightly higher value of plasma renin. This also invalidates the results shown in Figures 4 and 5. A higher number of mice is needed. This mayor concern was not addressed.

Answer: As indicated before, we consulted a statistician who advised us on the statistical analyses to be used for each experiment. As such, we found the statistically significant differences as presented, and we are convinced this represents true differences. Unfortunately we indeed observe quite some variation, but this makes it arguably even more impressive that we find a significant difference, in particular with tightly controlled physiological systems such as the RAAS.

Indeed, we were not able to increase group sizes (which would be beneficial for figure 5) but as previously indicated, although we aimed to perform additional mice experiments, unfortunately, due to strict legislative restrictions on animal experiments in the Netherlands, we are not allowed to do additional experiments unless we apply for a new permit, which takes 9 months on average. As such, doing new experiments would take at least a year, which would be, to our opinion, beyond a reasonable time. We do believe though that we provide ample *in vitro* and *in vivo* evidence for a regulatory role of miR-132 in fine-tuning renin levels.

3) As mentioned before, the statement that animals given "low salt diet in combination with antagomir-132 displayed a further increase in PGE2 and plasma renin levels" (Figure 5H and I) is based on a "trend" (p=0.06) for PGE2, that is PGE2 does NOT change. Based on your own data, the logical conclusion is that the increase in renin is independent of PGE2. In any case, the "n" needs to be increased. Furthermore, in the results section, you conclude that this data supports your hypothesis that the expression of miR-132 is salt-sensitive and serves as a negative feedback regulator of COX-2/PGE2-mediated renin synthesis. Please note that you are not showing data regarding renin synthesis or release by that matter: you are measuring steady-state renin levels in plasma. I am sorry about the limitations you may face at your institution, but research requires rigor, and the data should be convincing in order to be published.

Answer: As previously indicated, we do not see p-values around 0.05 as clear yes or no answers. I agree that based on P=0.06 we cannot definitely conclude there is a difference, but I believe it would also be wrong to conclude there is no difference. Most importantly, we do see a significant increase in (downstream) renin levels. Nonetheless, we agree we cannot draw strong conclusions on the PGE2 levels and have therefore changed our text describing that a trend is present but do not claim anymore there is a difference, while further studies would be necessary to yield more insight.

With regard to renin synthesis and/or release, this is a good point raised by the reviewer and this has been addressed as described under point 1).

4) The sample size for the in vitro experiments is not acceptable. For example, as stated in Figure 2B, n=4-6 (independent wells) is still n=1. All the wells are "independent". You need to average all the independent wells and repeat the whole experiment at least two extra times to have an n=3.

Answer: We agree that independent wells within one experiment actually means n=1. We should have better described this particular experiment, because what we have done is n=3 independent experiments with two wells per condition per experiment (where we missed values in 1 experiment for sc pMIR). However, we plotted all wells from the 3 experiments (yielding 4 or 6 data points),

which led to the confusing description. We should have plotted the averages of the 3 experiments, which we now have done in the novel figure 2B, as illustrated below.

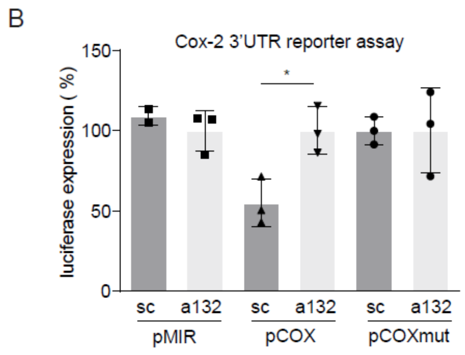

Novel Figure 2B.
